# Transient inhibition of sodium-glucose cotransporter 2 after ischemia/reperfusion injury ameliorates chronic kidney disease

**DOI:** 10.1172/jci.insight.173675

**Published:** 2024-03-22

**Authors:** Miguel Ángel Martínez-Rojas, Hiram Balcázar, Isaac González-Soria, Jesús Manuel González-Rivera, Mauricio E. Rodríguez-Vergara, Laura A. Velazquez-Villegas, Juan Carlos León-Contreras, Rosalba Pérez-Villalva, Francisco Correa, Florencia Rosetti, Norma A. Bobadilla

**Affiliations:** 1 Unidad de Fisiología Molecular, Instituto de Investigaciones Biomédicas, Universidad Nacional Autónoma de México, Mexico City, Mexico.; 2 Departmento de Nefrología y Metabolismo Mineral,; 3 Departmento de Fisiología de la Nutrición and; 4 Departmento de Patología Experimental, Instituto Nacional de Ciencias Médicas y Nutrición Salvador Zubirán, Mexico City, Mexico.; 5 Departmento de Biomedicina Cardiovascular, Instituto Nacional de Cardiología Ignacio Chávez, Mexico City, Mexico.; 6 Departmento de Inmunología y Reumatología, Instituto Nacional de Ciencias Médicas y Nutrición Salvador Zubirán, Mexico City, Mexico.

**Keywords:** Nephrology, Chronic kidney disease, Fibrosis, Mitochondria

## Abstract

Sodium-glucose cotransporter 2 (SGLT2) inhibitor, dapagliflozin (Dapa), exhibited nephroprotective effects in patients with chronic kidney disease (CKD). We assessed the efficacy of short-term Dapa administration following acute kidney injury (AKI) in preventing CKD. Male Wistar rats were randomly assigned to Sham surgery, bilateral ischemia for 30 minutes (abbreviated as IR), and IR + Dapa groups. Daily treatment with Dapa was initiated just 24 hours after IR and maintained for only 10 days. Initially, rats were euthanized at this point to study early renal repair. After severe AKI, Dapa promptly restored creatinine clearance (CrCl) and significantly reduced renal vascular resistance compared with the IR group. Furthermore, Dapa effectively reversed the mitochondrial abnormalities, including increased fission, altered mitophagy, metabolic dysfunction, and proapoptotic signaling. To study this earlier, another set of rats was studied just 5 days after AKI. Despite persistent renal dysfunction, our data reveal a degree of mitochondrial protection. Remarkably, a 10-day treatment with Dapa demonstrated effectiveness in preventing CKD transition in an independent cohort monitored for 5 months after AKI. This was evidenced by improvements in proteinuria, CrCl, glomerulosclerosis, and fibrosis. Our findings underscore the potential of Dapa in preventing maladaptive repair following AKI, emphasizing the crucial role of early intervention in mitigating AKI long-term consequences.

## Abstract 2

Dapaglifozin administration for 10-days after acute kidney injury prevents transition to chronic kidney disease by improving renal vascular resistance, NAD homeostasis, mitochondrial dynamics, and immune cell infiltration.

## Graphical abstract

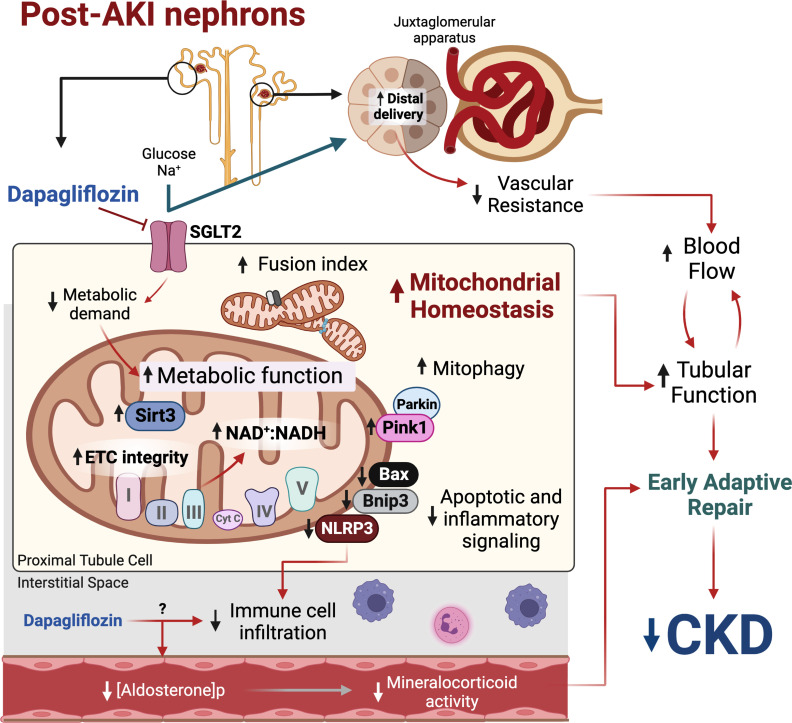

## Introduction

Acute kidney injury (AKI) is a clinical syndrome characterized by a reduction in kidney function established in less than 7 days that affects over 13 million people globally and contributes significantly to in-hospital mortality (1). Although most of AKI survivors recover adequate kidney function, it has been recognized that suffering an AKI episode increases the risk of late adverse outcomes, including rehospitalization, hypertension, cardiovascular events, higher mortality, and chronic kidney disease (CKD) (2, 3). Although the causal relationship between AKI and CKD is relatively obscure in the clinical setting, many research groups, including ours, have demonstrated that an experimental AKI episode is enough to lead to kidney dysfunction and fibrosis in the long-term through multiple maladaptive mechanisms (4–9). Since there is no current treatment intended to reduce this AKI-to-CKD transition, research efforts are urgently needed in this field, especially considering that most AKI cases are difficult to anticipate.

Although AKI includes patients with highly heterogenous pathophysiological processes, most cases involve a transient reduction in renal perfusion with a resulting ischemic insult against endothelial and tubular cells (1, 4). For this reason, ischemia/reperfusion injury (IRI) remains a relevant model to study AKI and its consequences. We have consistently demonstrated that a single episode of severe IRI leads to CKD in male Wistar rats, and this has allowed us to study different pathophysiological aspects of the AKI-to-CKD transition (5–9). Furthermore, we proved that transient therapeutical interventions in early stages of the disease, either before or shortly after AKI, can successfully prevent CKD in this model (8–10). Although these were encouraging findings, we still need to explore additional therapeutic options before translating them into the clinical setting.

Sodium-glucose cotransporter 2 inhibitors (SGLT2i), commonly known as flozins, are drugs developed in the last decade for glycemic control in the type 2 diabetes mellitus (T2D) population that have consistently proven a positive effect on adverse cardiovascular and renal outcomes (11–13). Moreover, the late DAPA-CKD trial proved a 44% reduction in a composite of a sustained decline in kidney function, onset of end-stage kidney disease (ESKD), or death from renal causes in dapagliflozin (Dapa) users compared with placebo over a median follow-up of 2.4 years, specifically in patients with CKD with or without T2D (14). Although the specific mechanisms driving renal protection in these patients remain elusive, there is compelling evidence that flozins modulate the macula densa–tubuloglomerular feedback (MD-TGF) and the renin angiotensin aldosterone system (RAAS) activity, while inducing a metabolic flexibility that works conjunctively to reduce glomerular abnormalities and preserve tubular epithelial homeostasis in both diabetic and nondiabetic CKD patients (15, 16). Interestingly, these mechanisms are also known to participate in AKI and mediate part of the maladaptive response that leads to CKD transition (17, 18). In fact, there are several studies demonstrating a reduction of organ damage in nondiabetic rodents when flozins are administered before an ischemic insult by improving mitochondrial homeostasis in the heart, lungs, and kidneys (19–21). However, the effects of a short treatment with these drugs on maladaptive repair and their capacity to prevent CKD after the onset of AKI remains unclear.

In this study, we evaluated the effects of a 10-day treatment with Dapa after 24 hours of an episode of ischemic AKI in rats. We hypothesized that this early and transient treatment would suffice to reduce the progression to CKD through a positive effect on kidney hemodynamics, the intrarenal RAAS, and mitochondrial homeostasis maintenance.

## Results

### A 10-day treatment with Dapa improved renal function recovery, despite persistent tubular injury after IRI.

**Figure 1:**
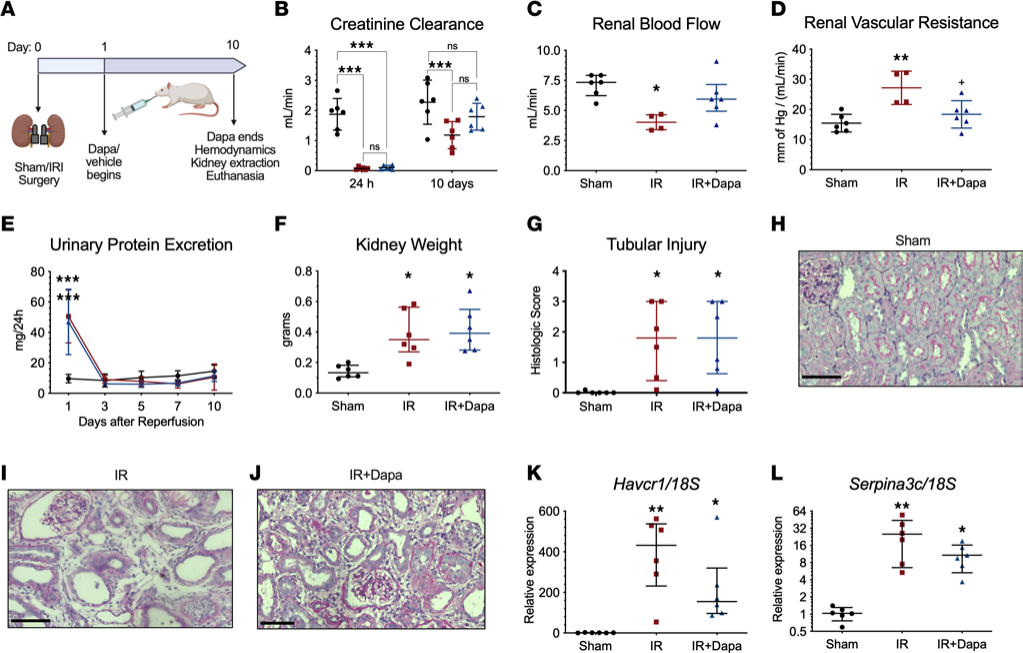
SGLT2 inhibition with dapagliflozin improved physiological renal recovery 10 days after AKI. (**A**) Experimental design (*n* = 18). (**B**) Creatinine clearance after 24 hours and after 10 days of reperfusion in the 3 studied groups: Sham (black circles), IR (red squares), and IR + Dapa (blue triangles). (**C**) RBF recordings at the tenth day (*n* = 4–6 per group). (**D**) RVR at the tenth day, calculated as the mean arterial pressure divided by RBF in each subject (*n* = 4–6 per group). (**E**) Serial urinary protein excretion in the 3 groups at 5 time points during the first 10 days of reperfusion (same color code apply). (**F**) Kidney weight 10 days after surgery. (**G**) Tubular injury score obtained in a blinded fashion; histological abnormalities included tubular simplification, dilation, basal membrane thickening, and interstitial hypercellularity. (**H**–**J**) Representative images showing renal morphology in PAS-stained slices of kidney cortex at ×200. Scale bar: 100 μm. (**K** and **L**) mRNA expression of 2 injury-related genes in renal cortex lysates including *Havcr1* and *Serpina3c*. *n* = 6 rats per group unless indicated. Statistical differences analyzed by Kruskal-Wallis test for **C**, **F**, and **K** (median ± IQR) and ANOVA F-test for the rest of the graphs (mean ± SD). *P < 0.05, **P < 0.01, ***P < 0.001 versus Sham; ^+^*P* < 0.05 versus IR.

Eighteen male Wistar rats were subjected to Sham surgery or 30 minutes of ischemia/reperfusion (IR). Twenty-four hours after surgery, AKI rats were randomized to receive either placebo (IR) or Dapa (IR + Dapa) for 10 days (Figure 1A). The effective delivery of the drug is indicated by prominent glucosuria and increased natriuresis (Table 1). After this period, mean arterial pressure (MAP), renal blood flow (RBF), and creatinine clearance (CrCl) were measured to evaluate renal functional recovery. Although both groups subjected to AKI exhibited a significant recovery of CrCl after 10 days compared with 24 hours of evaluation — there was a persisting mean reduction of –1.09 mL/min in IR compared with the Sham group (95% CI, 0.43–1.75; *P* = 0.007) — such an effect was improved with Dapa treatment (Figure 1B). Remarkably, we found a significant reduction in RBF corresponding with an increased renal vascular resistance (RVR) in the IR group, which was prevented with Dapa treatment (Figure 1, C and D). There were no differences in MAP values between groups (107 ± 6.2, 108 ± 6.1, and 104 ± 6.8 mm of Hg in Sham, IR, and IR + Dapa, respectively). These data support prior evidence that suggests that SGLT2 inhibition promotes a postglomerular vasodilatory response rather than a preglomerular vasoconstriction in the context of an already increased RVR (22), as seen here.

After 24 hours of reperfusion, the IR and IR + Dapa groups exhibited the characteristic increase in proteinuria (UprotV) induced by IRI, considering that neither group received any treatment at this time (Figure 1E). UprotV returned to basal levels, since the third day following IRI, and persisted normal even at the tenth day, without any difference among the groups (Figure 1E). This supports our previous findings showing that UprotV is not an early biomarker of maladaptive repair (23). At the tenth day and after measuring renal hemodynamic parameters, kidneys were harvested for histological and molecular evaluation. Both the IR and IR + Dapa groups showed a significant increase in kidney weight by ~2-fold compared with the Sham group (Figure 1F), which was associated with clear histological abnormalities including tubular dilation, basal membrane thickening, interstitial expansion, and hypercellularity in the kidney cortex (Figure 1, G–J). In addition, we found a significant increase in tubular injury–related mRNA levels, specifically *Havcr1* and *Serpina3c* in the IR group; although there was a trend of lower mRNA levels of these tubular injury biomarkers in the IR + Dapa group, they remained higher compared with the Sham group, indicating a persistent degree of tubular injury in both AKI groups (Figure 1, K and L).

### Effects of IR and Dapa on the intrarenal RAAS and other vasoactive molecules 10 days after reperfusion.

**Figure 2:**
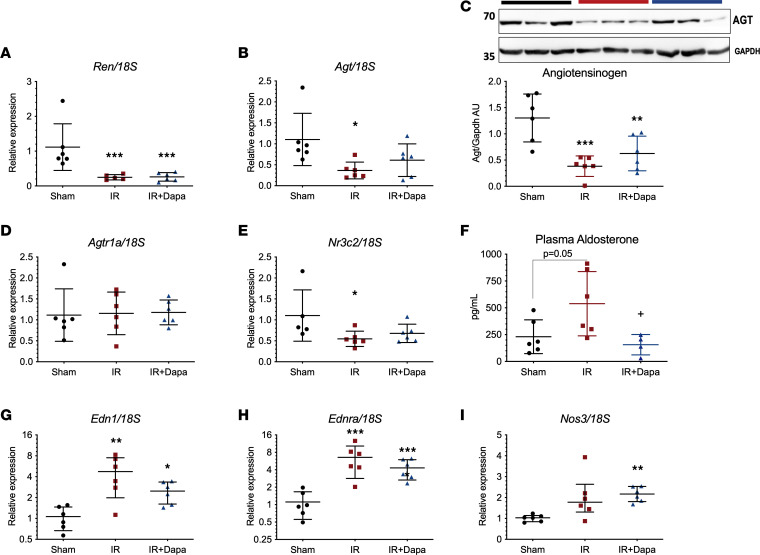
mRNA levels of intrarenal components of the renin-angiotensin system, plasma aldosterone concentration, and other vasoactive factors after 10 days of AKI and dapagliflozin treatment. (**A**–**E**) Intrarenal expression of the RAS components in the 3 groups: Sham (black circles), IR (red squares), IR + Dapa (blue triangles), including renin mRNA (*Ren*) (**A**), angiotensinogen mRNA (*Agt*) (**B**), Agt protein (**C**), the angiotensin-II type 1 receptor mRNA (*Agtr1a*) (**D**), and the mineralocorticoid receptor mRNA (*Nr3c2*) (**E**). (**F**) Plasma aldosterone concentration determined by immunoassay. (**G**–**I**) Expression of other vasoactive molecules, including endothelin 1 mRNA (*Edn1*) (**G**), the endothelin 1 receptor A mRNA (*Ednra*) (**H**), and the endothelial nitric oxide synthase mRNA (*Nos3*) (**I**). *n* = 6 rats per group. Statistical differences analyzed by ANOVA F-test for **A**–**H** (mean ± SD) and Kruskal-Wallis test for **I** (median ± IQR). Data in **A**, **B**, **D**, **E**, **G**, and **H** were log-transformed for analysis. *P < 0.05, **P < 0.01, ***P < 0.001 versus Sham; ^+^*P* < 0.05 versus IR.

The kidney expresses all the components of the RAAS system, which is a known mediator of local injury in different pathological settings (24). Considering the hypothesized effect of flozins on RAAS activation, we assessed the mRNA levels of renin (*Ren*), angiotensinogen (*Agt*), the angiotensin II type 1 receptor (*Agtr1a*), and the mineralocorticoid receptor (*Nr3c2*), as well as Agt protein, to evaluate the intrarenal RAAS in kidney cortex of the studied groups. Strikingly, we found a reduction of *Ren*, *Agt*, and *Nr3c2* expression in both IR and IR + Dapa groups (Figure 2, A–E). Additionally, AGT protein expression was also reduced in both AKI groups (Figure 2C). Interestingly, we found a significant increase of plasma aldosterone concentration in the IR group compared with Sham and IR + Dapa groups (538.4 versus 230.6 and 155.9 pg/mL, respectively; *P* = 0.03) (Figure 2F). Despite no statistical differences in plasma electrolyte levels among groups, the IR group exhibited greater values of plasma potassium than the Sham and IR + Dapa groups (4.7 versus 4.0 and 4.1 mEq/L, respectively; Table 1). Therefore, a potassium-dependent regulation of aldosterone cannot be ruled out, considering that the adrenal glands respond to minimal changes in circulating potassium levels. Additionally, endothelin 1 (*Edn1*) and its receptor type A (*Ednra*) transcripts were evaluated, because both are induced by IRI and aldosterone and may contribute to the sustained vasoconstriction after AKI (25); the expression of the endothelial nitric oxide synthase (eNOS or *Nos3*) as a central molecule in endothelial-induced vasodilation was also assessed. After AKI, there was a significant increase in both *Edn1* and *Ednra* mRNA levels, such effect was slightly reduced with Dapa treatment, together with an overexpression of *Nos3* mRNA levels even compared with the Sham group (Figure 2, G–I). These findings suggest that aldosterone, Edn1, and reduced eNOS may contribute to the renal vasoconstriction observed in early stages after AKI, with an improvement when Dapa is administered.

### Dapa improved ultrastructural abnormalities in proximal tubules and restored mitochondrial dynamics and turnover 10 days after AKI.

**Figure 3:**
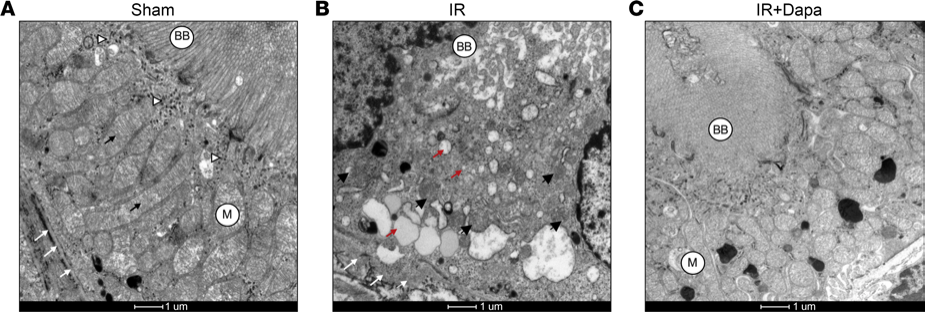
Ten days after AKI, dapagliflozin abrogates ultrastructural abnormalities in proximal tubules. Representative images taken through transmission electron microscopy. (**A**) Proximal tubular cell from a Sham rat exhibiting a characteristic morphology, with a tall apical brush border (BB), a prominent endocytic-lysosomal machinery (white arrowheads), long-shaped mitochondria (M) with conserved cristae (black arrows), and a regular basement membrane (white arrows). (**B**) Proximal tubular cell from an IR rat showing the typical loss of BB, abundant multisized vesicles (red arrows), small, round mitochondria (black arrowhead), and an irregular thickened basement membrane (white arrows). (**C**) Proximal tubular cell in an IR + Dapa rat showing structural similarities with **A**, a prominent BB, absence of anomalous vesicles, and conserved mitochondria.

Although the histologic abnormalities persisted in both AKI groups, we observed a mild restoration of tubular morphology in the treated animals when compared subjectively to the IR group (Figure 1, I and J). To follow up this issue, we evaluated the ultrastructural changes through transmission electron microscopy (TEM), focusing specifically on the proximal tubules. We found that Dapa significantly mitigated AKI-induced brush border loss, cytosolic vacuolation, and mitochondrial abnormalities, suggesting a greater response of this nephron segment to treatment (Figure 3). This observation aligns with the changes seen in *Havcr1* (Figure 1K). A striking observation was the presence of smaller, round-shaped mitochondria in the IR group compared with the Sham group (Figure 3, A and B), while the IR + Dapa group exhibited a network of hyperfused mitochondria (Figure 3C).

**Figure 4:**
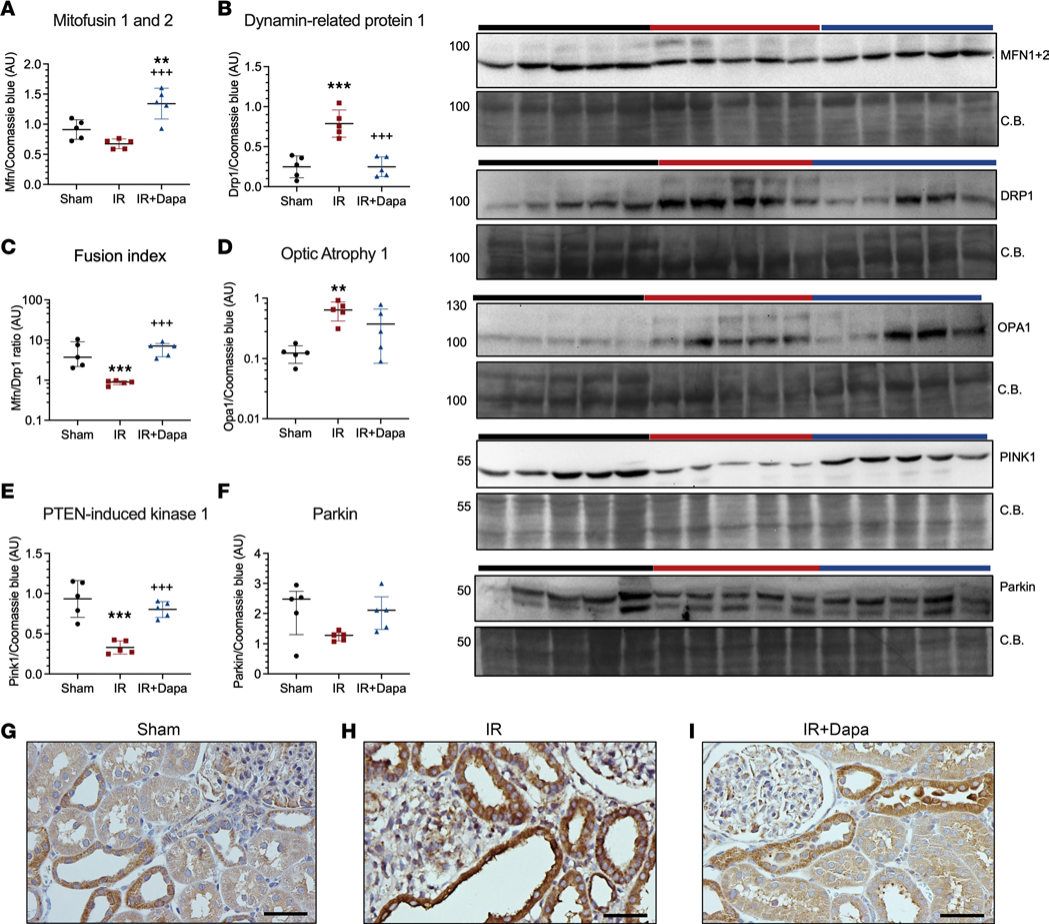
Ten days following AKI, dapagliflozin preserves mitochondrial dynamics and clearance. (**A**–**F**) Densitometric analysis for the expression of fusion/fission and mitophagy-related proteins in mitochondrial extracts from the renal cortex of the 3 groups: Sham (black circles), IR (red squares), IR + Dapa (blue triangles). (**A**) The outer mitochondrial membrane (OMM) proteins Mitofusin 1 and 2 (MFN). (**B**) The OMM Dynamin-related protein 1 (DRP1). (**C**) Fusion index calculated as the ratio between MFN and DRP1. (**D**) The inner mitochondrial membrane protein Optic Atrophy 1 (OPA1). (**E** and **F**) Expression of PTEN-induced kinase 1 (PINK1) and its effector Parkin. (**G**–**I**) IHC microphotographs indicating the expression of DRP1 in the 3 groups. *n* = 5 per group. Scale bar = 50 µm. Statistical differences were analyzed by ANOVA F-test for **A**–**F** (mean ± SD). Data in **C** and **D** were log-transformed for analysis. **P < 0.01, ***P < 0.001 versus Sham; ^+++^*P* < 0.001 versus IR. CB, Coomassie blue staining.

Given the significant role of mitochondrial injury and metabolic dysregulation in maladaptive repair and fibrosis following AKI, we conducted a comprehensive assessment of protein expression related to various aspects of mitochondrial homeostasis. This analysis was performed through Western blot on mitochondria isolated from the kidney cortex of the studied groups. First, we evaluated the regulators of outer mitochondrial membrane (OMM) fusion and fission: mitofusin 1 and 2 (MFN), and the dynamin-related protein 1 (DRP1). There was an increase in the expression of MFN in the IR + Dapa group compared with the other 2 groups (Figure 4A). Furthermore, there was a significant increment in DRP1 mitochondrial expression in the IR group compared with the Sham group, but this rise was not evident in the IR + Dapa group (Figure 4B); this was further corroborated through IHC, revealing an enrichment of this protein specifically in the injured tubules, as illustrated in Figure 4, G–I. Moreover, the MFN/DRP1 ratio indicated dysregulated mitochondrial dynamics that favor fission against fusion in the IR group, while Dapa induced the highest fusion index of all groups (Figure 4C), supporting the ultrastructural findings previously described. The expression of Optic atrophy 1 (OPA1), involved in the inner mitochondrial membrane (IMM) fusion and cristae remodeling, was also assessed. Unexpectedly, we found an upregulation of this protein in the IR group compared with the Sham, with a similar behavior in the treated animals (Figure 4D), which might indicate a compensatory response after AKI.

To assess the mitochondrial turnover capacity, the expression of the mitophagy-related proteins PTEN-induced kinase 1 (PINK1) and its effector, ubiquitin ligase Parkin, were assessed. Interestingly, we found that Dapa significantly prevented the downregulation of PINK1 seen in the IR group, with a similar pattern in Parkin (Figure 4, E and F), indicating that SGLT2 inhibition with Dapa restores the disrupted mitochondrial turnover that occurs after AKI.

### Dapa improved mitochondrial redox balance and metabolic dysfunction 10 days after AKI.

**Figure 5:**
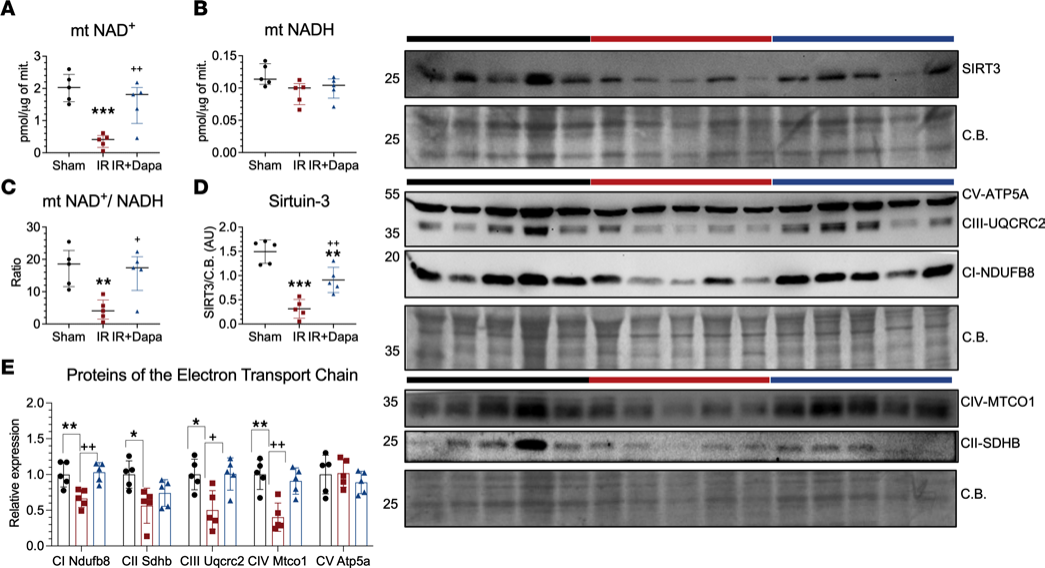
Ten days following AKI, dapagliflozin optimizes mitochondrial metabolic and redox response. (**A**–**C**) Colorimetric quantification of NAD species in mitochondrial extracts from the renal cortex in the 3 groups: Sham (black circles), IR (red squares), IR + Dapa (blue triangles). (**D**) Deacetylase Sirtuin-3 expression evaluated by Western blot. (**E**) Expression of subunits of the electron transport chain (ETC): complex I NDUFB8, complex II SDHB, complex III UQCRC2, complex IV MTCO1, and complex V ATP5A from the ATP-synthase. *n* = 5 rats per group. Statistical differences were analyzed by ANOVA F-test for **A**–**E** (mean ± SD). *P < 0.05, **P < 0.01, ***P < 0.001 versus Sham; ^+^*P* < 0.05, ^++^*P* < 0.01 versus IR. CB, Coomassie blue staining.

To deepen into mitochondrial dysfunction in the studied groups, we evaluated the mitochondrial nicotinamide adenine dinucleotide (mtNAD) balance and the integrity of proteins within the electron transport chain (ETC) responsible for oxidative phosphorylation (OXPHOS). There was a significant recovery of mtNAD^+^ with a corresponding increase in the NAD^+^/NADH ratio in the IR + Dapa compared with the IR group, which showed a significant reduction in both parameters compared with the Sham group (Figure 5, A–C). Moreover, Dapa exhibited a mitigating effect on the downregulation of the mitochondrial deacetylase Sirtuin-3 observed in the IR group (Figure 5D). In addition, we observed a compromised ETC integrity in the IR group compared with the Sham group, with only the ATP5A subunit of complex V remaining unaffected at this time point (Figure 5E). Notably, Dapa played a crucial role in preserving the ETC, leading to restoration of complexes I, III, and IV to essentially normal levels (Figure 5E). These results strongly suggest that Dapa optimizes mitochondrial metabolic function and redox balance during the repair phase of AKI.

### Dapa improved the apoptotic and inflammatory mitochondrial signaling seen 10 days after AKI.

**Figure 6:**
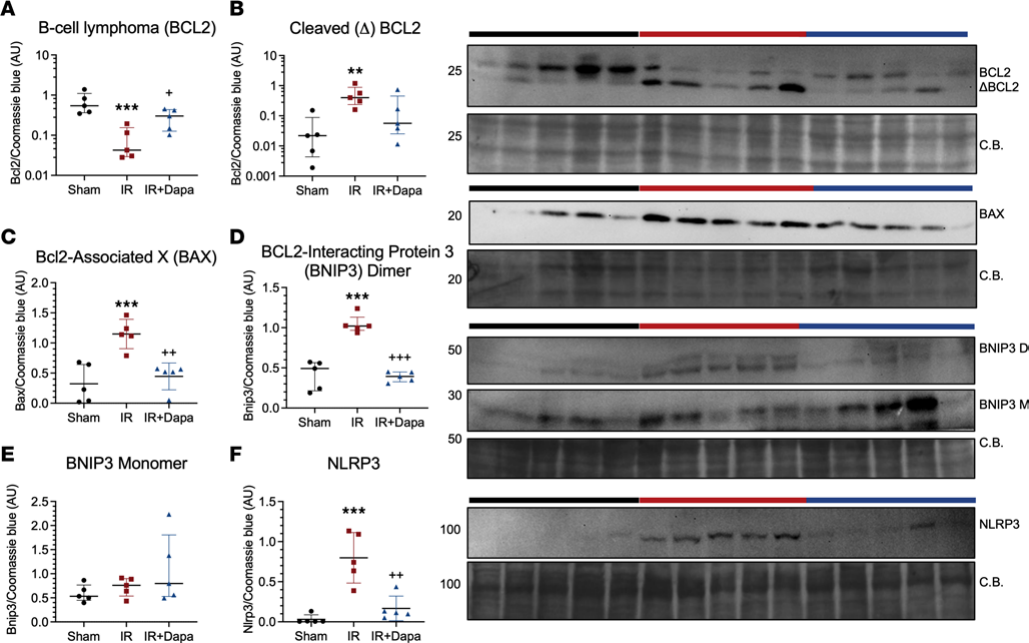
Ten days after AKI, dapagliflozin reduces mitochondrial proapoptotic and proinflammatory signaling. (**A**–**F**) Densitometric analysis for the expression of outer mitochondrial membrane signaling proteins in mitochondrial extracts from the renal cortex of the 3 groups: Sham (black circles), IR (red squares), IR + Dapa (blue triangles). (**A**) The apoptotic regulator B-cell lymphoma (BCL2). (**B**) Approximately 20 kDa cleaved product of BCL2. (**C**) The BCL2–associated X (BAX). (**D**) The BCL2–interacting protein 3 (BNIP3) dimer at ~55 kDa. (**E**) The BNIP3 monomer at ~30 kDa. (**F**) The inflammatory regulator NLR family pyrin domain containing 3 (NLRP3). *n* = 5 rats per group. Statistical differences were analyzed by ANOVA F-test for **A**–**F** (mean ± SD). **A** and **B** were log-transformed for analysis. **P < 0.01, ***P < 0.001 versus Sham; ^+^*P* < 0.05, ^++^*P* < 0.01, ^+++^*P* < 0.001 versus IR. CB, Coomassie blue staining.

Beyond their involvement in cell metabolism and bioenergetics, mitochondria play a central role in cellular signaling. Specifically, the OMM serves as a platform for proteins that regulate cell death and inflammation, including members of the B cell lymphoma 2 (BCL2) family. Consistent with our previous findings, the IR group exhibited a reduction of the antiapoptotic protein BCL2 at its typical molecular weight (~28 kDa), accompanied by a distinct shift to ~20 kDa; this likely corresponds to a smaller, proteolytic product (Δ^1–34^ BCL2; see ref. 26) possessing proapoptotic activity (Figure 6, A and B) that was attenuated with Dapa treatment. Furthermore, an enrichment of the proapoptotic proteins BCL2–associated X (BAX) and the BCL2–interacting protein 3 (BNIP3) in its dimeric form (~50 kDa) was evident exclusively in the IR group (Figure 6, C–E). These findings indicate a persistent dysregulation in the control of apoptosis following AKI, a disturbance significantly mitigated by Dapa administration. Besides, there was a discernible enrichment of the NLR family pyrin domain containing 3 (NLRP3) protein, commonly referred to as the inflammasome, within the mitochondria of the IR group, in contrast to the absence of such enrichment in the other 2 groups (Figure 6F).

Given our findings in NLRP3, we assessed immune cell infiltration by flow cytometry (Supplemental Figure 1; supplemental material available online with this article; https://doi.org/10.1172/jci.insight.173675DS1). An increase in leukocyte infiltration in the renal cortex of the IR group was observed (Supplemental Figure 2A), involving myeloid (neutrophils and macrophages) and lymphoid lineages (T cells). Consistent with previous studies, the IR + Dapa group exhibited a reduction in proinflammatory CD11b^+^ myeloid cells compared with the IR group, while a modest effect on CD3^+^ T lymphocyte infiltration was observed (Supplemental Figure 2). Further differentiation of M1 and M2 subpopulations within CD68^+^ cells was carried out based on CD11b positivity (intermediate versus high) and assessing CD86 and CD206 markers (Supplemental Figure 1). This analysis revealed a blunted increase of M1 cells in the IR + Dapa group compared with the IR group, without significant changes in the M2 subpopulation between these groups (Supplemental Figure 2, D and E). At this time point, we also observed a significant increase in the expression of acute-phase proinflammatory and late-phase profibrotic cytokines in the IR group compared with the Sham group, but there was a consistent trend indicating a mild reduction in all these molecules after SGLT2 inhibition (Supplemental Figure 3). Taken together, these findings support an optimized resolution of inflammation with Dapa during postischemic renal repair, despite suffering the same AKI severity.

### Dapa partially improved mitochondrial dysfunction 5 days following AKI, before hemodynamic recovery.

**Figure 7:**
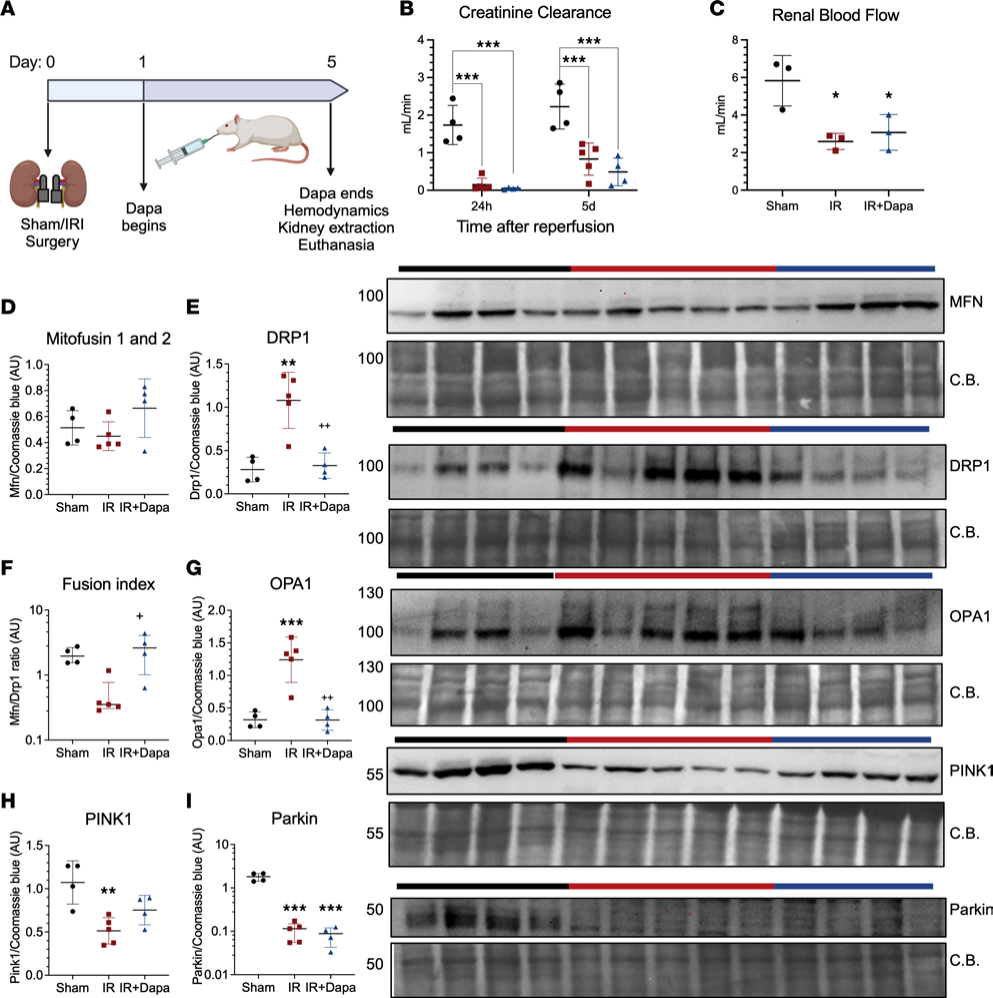
Five days following AKI, dapagliflozin optimizes restoration of mitochondrial dynamics prior to recovery of renal function. (**A**) Experimental design (*n* = 13). (**B**) Creatinine clearance at 24 hours and 5 days after surgery. Sham (black circles), IR (red squares), and IR + Dapa (blue triangles) are shown. (**C**) Renal blood flow at day 5 (*n* = 3 per group). (**D**–**I**) Densitometric analysis of protein expression of regulators of mitochondrial dynamics and clearance: Mitofusin 1 and 2 (MFN) (**D**), DRP1 (**E**), fusion index calculated as in Figure 4 (**F**), OPA1 (**G**), PINK1 (**H**), and Parkin (**I**) and their respective representative Western blot images (**J**). Statistical differences were analyzed by ANOVA F-test in all panels. Data in **F** and **I** were log transformed for analysis. *n* = 4–5 per group unless indicated. **P < 0.01, ***P < 0.001 versus Sham; ^+^*P* < 0.05, ^++^*P* < 0.01 versus IR. CB, Coomassie blue staining.

**Figure 8:**
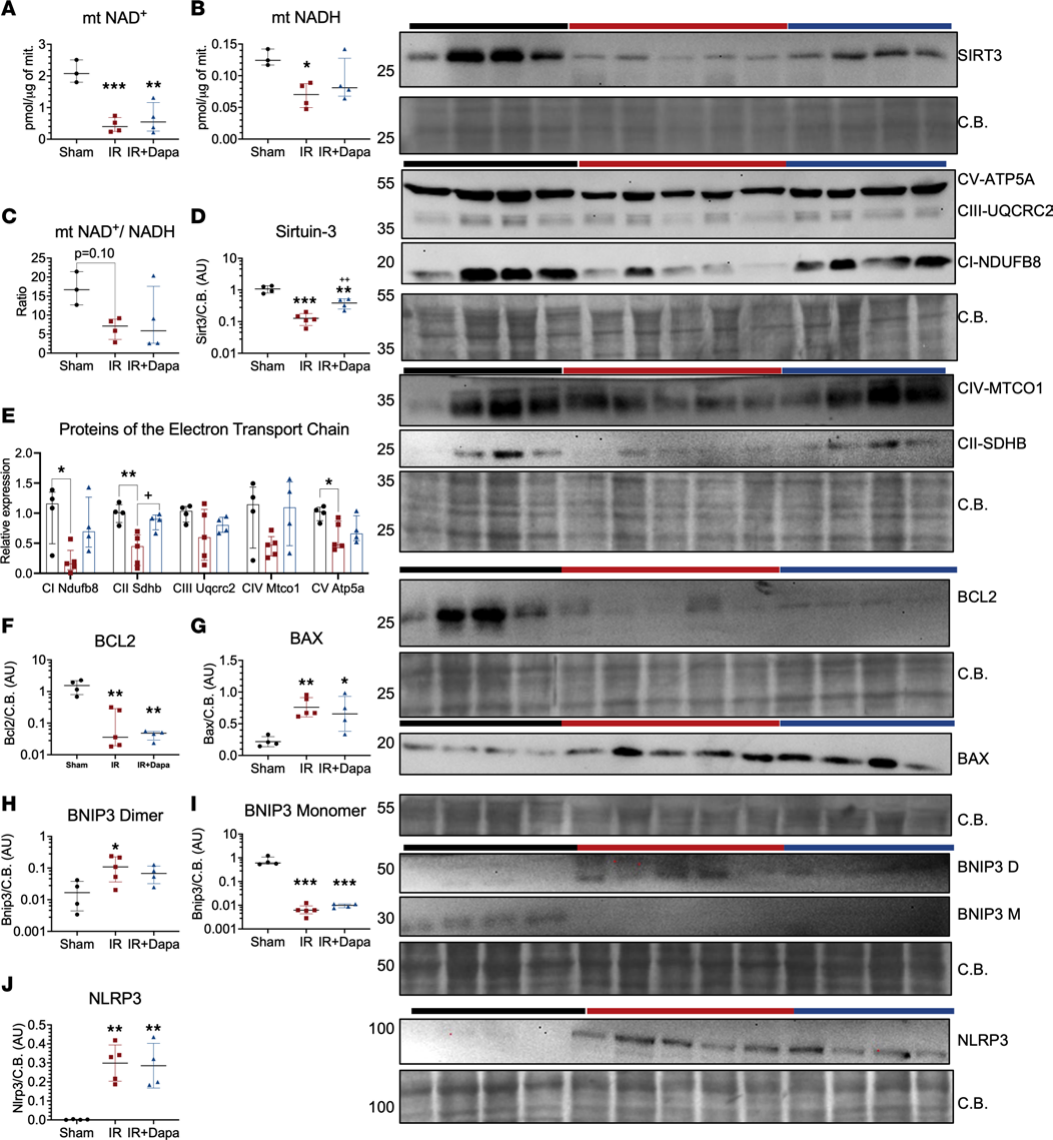
Five days following AKI, dapagliflozin reduces mitochondrial metabolic dysfunction but not abnormal outer membrane signaling. (**A**–**C**) Colorimetric quantification of NAD species in mitochondrial extracts from the renal cortex in the 3 groups (*n* = 3–4 per group). Sham (black circles), IR (red squares), and IR + Dapa (blue triangles) are shown. (**D**–**I**) Densitometric analysis of mitochondrial protein expression through Western blot: Sirtuin-3 (**D**), subunits of the electron transport chain (ETC) as in Figure 4 (**E**), BCL2 (**F**), BAX (**G**), BNIP dimer (**H**), BNIP3 monomer (**I**), and NLRP3 (**J**). Statistical differences analyzed by ANOVA F-test for all panels. Data in **D**, **F**, **H**, and **I** were log transformed for analysis. *P < 0.05, **P < 0.01, ***P < 0.001 versus Sham; ^+^*P* < 0.05, ^++^*P* < 0.01 versus IR. CB, Coomassie blue staining.

To gain a more comprehensive understanding of the effect of Dapa on mitochondrial homeostasis after AKI, an additional set of rats was included. These rats underwent the same group assignment but were euthanized 5 days after IRI (Figure 7A). Notably, both AKI groups exhibited a persistent decline in renal function, as evidenced by a significant reduction in CrCl and in RBF when compared with the Sham group (Figure 7, B and C). Surprisingly, our findings revealed that Dapa significantly prevented the upregulation of DRP1 and OPA1 already observed in the IR group at this time point. In contrast, the treatment had minimal effect on PINK1 and Parkin, which were significantly reduced in both AKI groups compared with the Sham group (Figure 7, D–J). Despite the fact that both AKI groups displayed a comparable reduction in NAD species compared with the Sham group, we found a partial beneficial effect of Dapa on the ETC proteins and SIRT3 even at this time point of repair (Figure 8, A–E). Finally, we observed similar changes in BCL2, BAX, BNIP3, and NLRP3 in both AKI groups, suggesting that treatment has not yet attenuated proapoptotic and inflammatory signaling as seen in the 10-day cohort (Figure 8, F–J). Collectively, these findings suggest that Dapa contributes to a partial protection of mitochondrial homeostasis during the initial phases of maladaptive repair following AKI. This effect is noteworthy, occurring even before the recovery of renal hemodynamics has taken place.

### Transient treatment with Dapa reduced CKD development 5 months after AKI.

**Figure 9:**
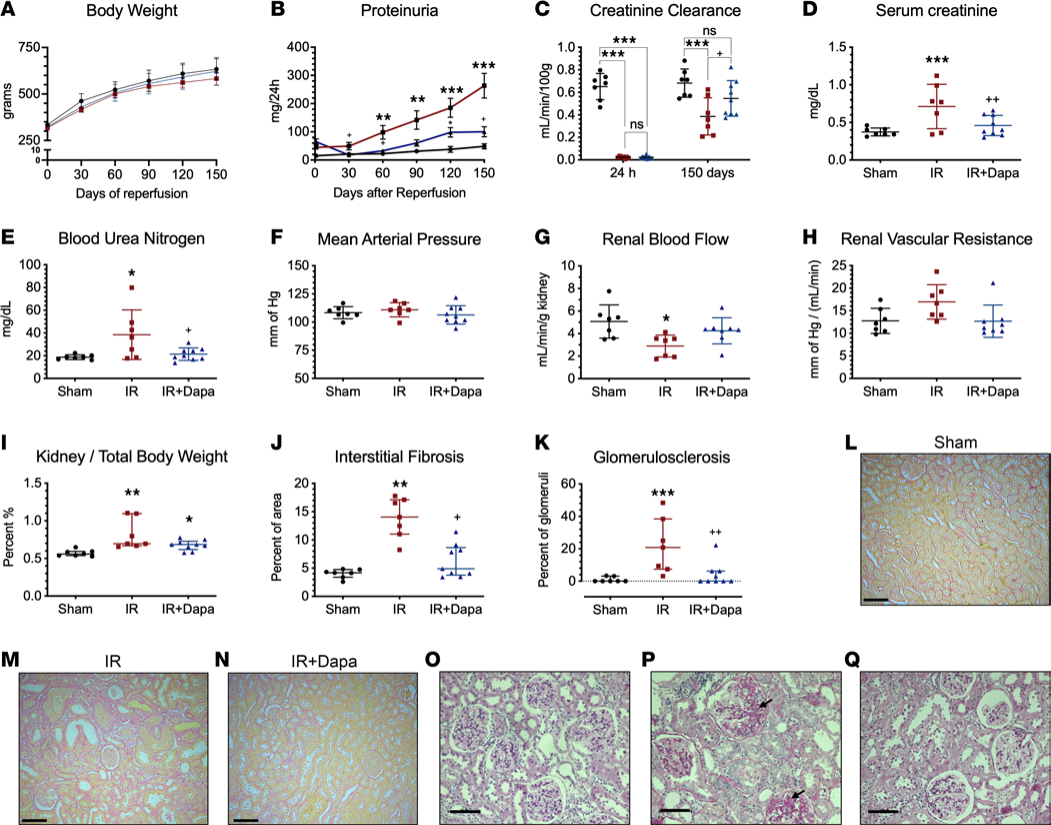
A 10-day treatment with dapagliflozin after IRI prevented AKI-to-CKD transition. CKD phenotype was evaluated in an independent cohort with the same experimental design. Sham (black circles), IR (red squares), and IR + Dapa (blue triangles) are shown. (**A**) Time-related increase in body weight in the 3 groups (mean ± SD) (**B**) Progressive development of proteinuria after the initial surgery, in the 3 groups (mean ± SEM; multiple comparisons described in Supplemental Table 1). (**C**–**E**) Kidney function evaluated by CrCl, serum Cr, and BUN levels, respectively. (**F**–**H**) Hemodynamic changes in MAP, RBF, and RVR. (**I**) Kidney/body weight ratio to evaluate renal hypertrophy. (**J**) Histological analysis of interstitial fibrosis evaluated through Sirius red staining. (**K**) Histological analysis of glomerulosclerosis (GS) evaluated in PAS-stained slices. (**L**–**N**) Representative images showing Sirius red–stained cortex slices (×150). Scale bar: 100 μm. (**O**–**Q**) Representative images showing GS (arrows) evaluated in PAS-stained slices (×200). Scale bar: 100 μm). ANOVA F-test was used for **C**–**H** (mean ± SD). Kruskal-Wallis test was used for **I**–**K** (median ± IQR). *n* = 7–9 per group. *P < 0.05, **P < 0.01, ***P < 0.001 versus Sham; ^+^*P* < 0.05, ^++^*P* < 0.01 versus IR.

Based in the previous results, we concluded that SGLT2 inhibition with Dapa favors a better adaptive kidney repair after 10 days of an ischemic AKI episode. To assess the long-term effects of this intervention, an independent set of rats with the same group assignment was studied, Dapa treatment was stopped on day 10, and rats were followed for 5 months to let CKD progress. A similar increase in body weight over time in all animals was found (month factor, *P* < 0.001), regardless of group assignment (treatment factor, *P* = 0.24) (Figure 9A). To assess the temporal variation in UprotV across groups, we employed linear mixed models, followed by Bonferroni post hoc pairwise comparison (Table 2 and Supplemental Table 2). The analysis revealed a significant difference among the groups after adjusting for months (*P* < 0.001). Notably, we found a blunted increase in UprotV after SGLT2 inhibition, with an evident difference between the IR and IR + Dapa groups at the end of the study (263.5 versus 100.2 mg/d; *P* = 0.02) (Figure 9B and Supplemental Table 2).

As expected, the IR group displayed renal dysfunction, characterized by a decrease in CrCl and elevated BUN levels compared with the Sham group. Conversely, the IR + Dapa group demonstrated a preservation of kidney function even at this late stage, also preventing the mild changes in plasma electrolytes seen in the IR group (Figure 9, C–E, and Table 3). Hemodynamically, no significant changes were observed in MAP among groups (Figure 9F), aligning with our previous reports indicating the absence of hypertension in this model of CKD (5, 8–10, 27). However, there was a significant reduction in RBF in the IR group compared with the Sham group, consistent with a trend toward a higher RVR, which was attenuated in the IR + Dapa group (Figure 9, G and H). Interestingly, both AKI groups exhibited a slight increase in the kidney/body weight ratio in Figure 9I, consistent with the early renal hypertrophy observed at day 10 regardless of Dapa administration (Figure 1F). Nevertheless, it is noteworthy that short-term Dapa treatment resulted in a significant reduction of the interstitial fibrosis and glomerulosclerosis seen in the IR group (Figure 9, J–Q). These findings confirm the establishment of nephroprotection, evident from the early phases of kidney repair.

## Discussion

**Figure 10:**
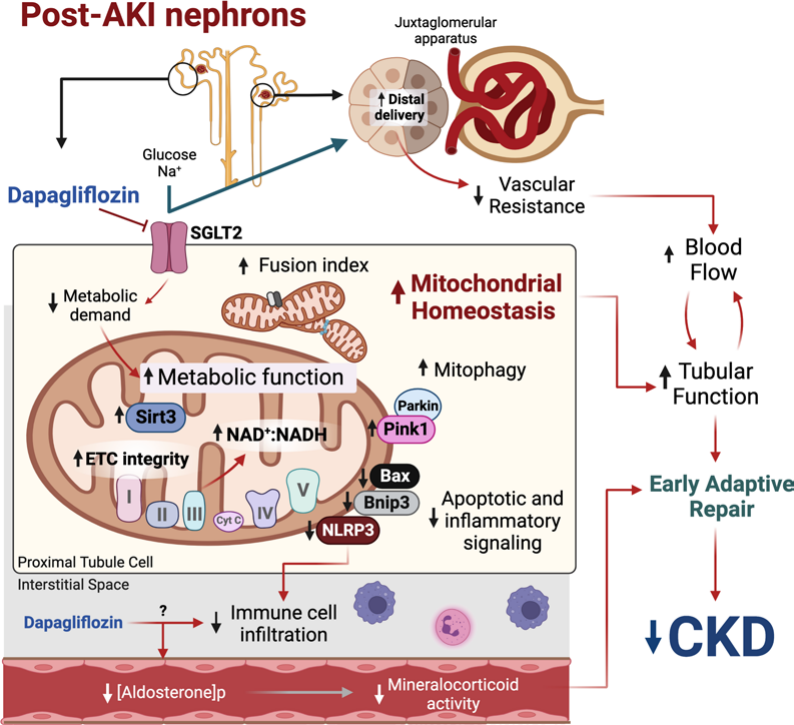
Proposed model of the mediators of dapagliflozin-induced adaptive repair after AKI. SGLT2 inhibition after ischemic injury reduces metabolic demand in the proximal tubules and improves mitochondrial metabolic function, which is accompanied by increased fusion, mitophagy, and reduced abnormal signaling from these organelles. A reduction in inflammatory infiltration may result directly from the drug and indirectly from tubular recovery. Increased distal fluid delivery results in reduced vascular resistance, which contributes to blood flow recovery and helps to restore tubular function. Treatment also reduces postischemic plasma aldosterone levels. All of these mechanisms establish early adaptive repair and prevent late chronic kidney disease (CKD). ETC, electron transport chain. Created with BioRender.com.

In the last decade, the medical community has been amazed by the beneficial effects of flozins in patients with T2D, heart failure, and CKD (13, 14, 28). Despite this compelling clinical evidence, the mechanisms behind these benefits remain elusive, which prevents a better rational guidance in flozins indication according to different clinical settings and represents an obstacle to the development of more specific therapeutic agents. In the present study, we demonstrated that a transient administration of Dapa reduces relevant hallmarks of maladaptive repair after a severe episode of AKI, including restoration of RVR and aldosterone concentration, maintenance of mitochondrial homeostasis, and abrogation of renal inflammation (Figure 10). More importantly, this short treatment was sufficient to reduce CKD development after 5 months of AKI, highlighting the translational implications of our findings. In fact, we are currently enrolling patients to receive Dapa shortly after severe AKI to assess the effect on CKD development and other major adverse kidney events (NCT05713851).

The acute drop in eGFR observed in patients with T2D and CKD after the initiation of flozins has been postulated as the central mechanism of nephroprotection (15, 16, 29). This effect is mediated by reduced sodium-glucose uptake in the proximal tubule, leading to increased fluid delivery to the distal nephron, which promotes activation of the MD-TGF. This system regulates glomerular hemodynamics in a chloride-dependent manner (30), involving various chemical mediators such as adenosine and ATP (31, 32), which induce afferent vasoconstriction and efferent vasodilation with consequent reduction in glomerular capillary pressure and ultrafiltration. Essentially, the net effect on afferent resistance has been worth noting in studies focusing on diabetic hyperfiltration and SGLT2 inhibition (31), while efferent arteriolar vasodilation has been neglected. Interestingly, recent clinical and experimental evidence suggests that postglomerular regulation of RVR also contributes to the effects of flozins (22, 33), becoming even more relevant in the context of increased afferent vasoconstriction. After IRI, there is a reduction in the diameter of the afferent arteriole and a dysfunction of its autoregulatory response, which, in addition to the increased activity of vasoactive molecules, leads to persistent hypoperfusion, even several days after AKI (9, 34). In this context, it is reasonable to conclude that Dapa reduced RVR through an early postglomerular vasodilation that improved microcirculation and tubular recovery after AKI.

Another relevant effect of the MD-TGF is the downregulation of renin release from the granular cells in the afferent arteriole, which regulates angiotensin II and aldosterone levels (35). The intrarenal RAAS has been recognized as a contributor to kidney pathophysiology, and its local regulation is relatively independent of the circulating axis (24, 36). In particular, there is evidence that angiotensin II levels increase 24 hours after IRI and that urinary Agt can be used as a biomarker of kidney injury, reflecting its intrarenal production and activity (37–39). Indeed, we previously showed that administration of losartan prior to IRI did not prevent AKI but abrogated CKD progression in rats, supporting a role for angiotensin II type 1 receptor (AT_1_) signaling in AKI repair (9). In contrast, another study reported a reduction in renin, ACE, ACE2, and AT_1_ mRNA levels, just 4 hours after unilateral IRI (40). These findings suggest a dynamic regulation of the intrarenal RAAS depending on the time point after AKI. Although some authors have speculated a downregulation of the RAAS during SGLT2 inhibition in diabetes (15), this has not been supported by clinical data, which even suggest an increase in circulating RAAS activity as a consequence of the diuretic effect of flozins (22, 41). Surprisingly, we observed a reduction in renin, AGT, and the *Nr3c2* mRNA levels, 10 days after reperfusion in both the IR and the IR + Dapa groups. However, plasma aldosterone increased > 2-fold after AKI, which was prevented by Dapa. During AKI, tubular dysfunction leads to electrolyte abnormalities, and hyperkalemia in particular can induce aldosterone secretion from the adrenal glands in a sensitive voltage-dependent manner (42). Although we did not observe statistical differences in potassium levels among groups, there was a slight increase in the IR group but not so in the IR + Dapa group. We speculate that the observed changes in aldosterone are the result of a greater functional recovery of the tubular epithelium and potassium homeostasis with Dapa. Moreover, the reduction in aldosterone may also directly contribute to adaptive renal repair, which is supported by our previous studies, where spironolactone reduced AKI-to-CKD transition in rats (8, 10).

Several studies have also demonstrated relevant effects on metabolism and on mitochondrial homeostasis with flozins. This has been attributed to a net urinary caloric loss that promotes a shift in fuel consumption in different organs (43). In this regard, Zhang et al. demonstrated that cytosolic glucose levels in the proximal tubules virtually disappeared when luseogliflozin was administered during reperfusion (44). Furthermore, a recent study by Billing et al. employing comprehensive multiomics technology showed that Dapa induces significant alterations in the early proximal tubule proteome. These alterations encompass numerous solute transporters within a complex interactome network, collectively leading to a reduced metabolic demand in this specific nephron segment (45). Our hypothesis centered around the notion that these changes would influence energetic balance and mitochondrial homeostasis during the repair process after AKI. An appropriate metric for assessing these parameters is the mtNAD^+^/NADH balance, which holds significance in maintaining redox balance, regulating numerous enzymes, and being sensitive to metabolic disruptions, including ischemic injury (46, 47). Notably, our findings reveal that Dapa had a restorative effect on the mtNAD^+^ pool and led to an increased NAD^+^/NADH ratio, compared with the IR group. Additionally, the treatment resulted in an upregulation of ETC proteins, particularly those within complexes I, III, and IV. This observation indicates a crucial recovery of mitochondrial metabolic function and redox balance with Dapa treatment. The significance of NAD metabolism in kidney heath and disease has been extensively discussed elsewhere (48). Of particular importance is the maintenance of the NAD^+^ pool through 3 major pathways in kidney cells. It may have implications for our findings: (a) the sodium-monocarboxylate transport (Preiss-Handler pathway), (b) the salvage pathway, and (c) de novo biosynthesis (49–51). It is noteworthy that the preservation of ETC integrity induced by Dapa in our study is likely associated with the restoration of the NAD^+^/NADH ratio, since these proteins favor the oxidized form of this coenzyme (47). Interestingly, these effects on the ETC were partially observed as early as the fifth day after AKI, even in the absence of NAD recovery, suggesting that the effect on ETC precedes the restoration of NAD^+^. Further investigation is needed to elucidate the mechanism underlaying NAD restoration during AKI and SGLT2 inhibition. Remarkably, NAD^+^ serves as a cofactor for enzymes that regulate mitochondrial homeostasis, including Sirt3. In fact, Sirt3 is necessary to maintain tricarboxylic acid cycle, β oxidation, the ETC, redox balance, and mitochondrial integrity in renal pathophysiology (52). Consistently, we found an upregulation of SIRT3 during SGLT2 inhibition 5 and 10 days following AKI, suggesting that this deacetylase may be an important mediator of the nephroprotective effects of Dapa seen in our study. The potential improvement in mitochondrial function with the restoration of RBF at day 10 is noteworthy. Equally significant is the observed preservation of SIRT3 and the ETC at day 5, even in the absence of hemodynamic recovery, suggesting an independent initiation of this mechanism by Dapa. Moreover, this independent initiation raises the intriguing possibility that it actively contributes to RBF autoregulation recovery.

Mitochondrial injury involves a loss of the OMM potential, making it a platform for the recruitment of several proteins that mediate fission, mitophagy, and cell death (53, 54). Accordingly, we observed an abnormal fusion/fission balance in the IR group, which was prevented in the treated animals on both days 5 and 10. This effect of flozins on mitochondrial dynamics has been reported also in diabetic kidney disease, indicating a consistent mechanism of nephroprotection (55). Unexpectedly, we also found an increase in OPA1, a mediator of the IMM fusion, after AKI at the same time points, and it was attenuated with Dapa administration at day 5. Importantly, there are physiological functions of OPA1 that extend beyond mitochondrial fusion, including cytochrome c sequestering, maintenance of energy balance, and mitochondrial DNA stabilization (56). Although this finding is initially counterintuitive, it may result as an adaptive response that attempts to counteract the rest of the mitochondrial abnormalities observed after AKI; however, further research is needed to confirm our findings and to elucidate the role of OPA1 in this model. On the other hand, we observed evidence indicating an abnormal clearance of damaged mitochondria after AKI. This inability to clear damaged mitochondria may be due to a reduction in PINK1 and Parkin, which facilitates the attachment of phagophores to the OMM of dysfunctional mitochondria. Interestingly, this was mitigated with Dapa at day 10, suggesting a role of this process toward nephroprotection. In addition, our results indicate that Dapa reduces the proapoptotic and proinflammatory signaling from mitochondria,as indicated by the observed changes in BCL2, BAX, BNIP3, and NLRP3 in the isolated organelles. Notably, these responses were not completely established by day 5, suggesting that they are either secondary to the metabolic recovery or require more treatment time to be induced by the drug; further research will shed light on these processes.

Finally, we also observed a reduction in renal inflammation with Dapa, as indicated by reduced myeloid infiltration, which was consistent with the reduction in mitochondrial NLRP (57). Although SGLT2 expression in leukocytes in vivo is unclear, there are studies suggesting a direct effect of flozins on monocytes and endothelial cells in models of ischemic myocardial injury and in hyperglycemic or septic in vitro assays (58–60). Interestingly, Dapa reduced TLR4 and NF-κB activation in differentiated macrophages and human endothelial cells stimulated with LPS (58). In addition, this drug also suppressed M1 and promoted M2 macrophage polarization in postischemic hearts, suggesting a downregulation of inflammation (59). Moreover, incubation with empagliflozin reduced oxidative stress in human monocytes and endothelial cells exposed to hyperglycemic conditions, resulting in reduced chemotaxis and endothelial dysfunction (60). Although the reduced leukocyte infiltration observed in the IR + Dapa group may be an indirect effect of improved tubular repair, we cannot exclude a direct regulation of proinflammatory signals in immune and endothelial cells leading to an earlier resolution of inflammation with Dapa.

In conclusion, our study contributes to the current understanding of the beneficial effects of SGLT2 inhibitors, and it specifically demonstrates that this therapy reduces maladaptive repair and CKD development, even when it is administered for only a short period after AKI has occurred. This highlights the importance of the early stages in determining long-term renal recovery and the importance of translating our findings into the clinical setting.

## Methods

### Sex and biological variables.

Sixty male Wistar rats weighting 300–350 g were included in the study. Our study exclusively examined male rats because the disease model AKI-to-CKD does not occur in females (5).

### Animal housing.

Sixty male Wistar rats weighting 300–350 g were included in the study. The animals were kept in a 12:12-hour day-night cycle, with controlled conditions of temperature and humidity in ventilated racks and with free access to food and water. For urine collection, rats were placed on metabolic cages for 18 hours with free access to water in time points specified below and returned to their cages until the end of the study.

### IRI model.

IRI was performed as previously described (9, 10). Briefly, rats were anesthetized with i.p. sodium pentobarbital (30 mg/kg) and placed on a heating pad for body temperature. The renal pedicles were accessed through a laparotomy. A 30-minute ischemic insult was induced by placing nontraumatic clamps on both renal pedicles; withdrawn, both ischemia and reperfusion were verified visually. Abdominal muscle and skin incisions were closed using vicryl and silk 3-0, respectively. For sham surgery, same procedures were performed, omitting pedicle clamping. Once awake, rats were placed in metabolic cages for urine collection. Twenty-four hours after surgery, 600 μL of blood were sampled from the lateral caudal vein using a heparinized 21-gauge needle. An overall mortality of 14% was observed within 120 hours of reperfusion only in rats subjected to IRI. Afterward, animals were followed for 5, 10, or 150 days as described below.

### Group assignment and Dapa administration.

Initially, 2 independent cohorts were included in our study, one followed for 10 days (short-term) after surgery and another for 150 days (long-term). In each cohort, rats were randomly assigned to 1 of 3 groups: sham-operated rats (S group), IRI-operated rats receiving 600 μL of 0.5% carboxymethyl-cellulose as vehicle (IR group), and IRI-operated rats receiving Dapa exclusively from day 1 to 10 after the surgery (IR + Dapa group). Dapa was prepared at a concentration of 0.5 mg/mL using 0.5% carboxymethyl-cellulose and administered through gastric gavage (1 mg/kg/d). In the long-term cohort, monthly urine collection was obtained to assess UprotV. An additional set of rats with the same group distribution was incorporated post hoc and studied for a shorter duration of 5 days. This addition was made to more closely examine the early response to treatment in AKI.

### Kidney functional studies.

Blood samples were centrifuged at 6,708*g* for 10 minutes at room temperature, and plasma was recovered and stored at –70°C until usage. After urine collection, samples were aliquoted and stored at –70°C until usage. Creatinine concentration was assessed through a modified rate Jaffé method, specifically suitable to mitigate potential bias, particularly when ketone bodies are of concern (A40920, Beckman Coulter), in automated equipment (UniCel DxC 600, Synchron Clinical System). CrCl was calculated using the typical formula CrCl = (Ucr × V)/Pcr, where Ucr represents urinary creatinine concentration (mg/dL), V represents urinary output (mL/min), and Pcr represents plasma creatinine concentration (mg/mL). Blood urea nitrogen and electrolytes were analyzed using the respective general chemistry reagents from Beckman Coulter. UprotV was determined by the turbidimetric method using trichloroacetic acid precipitation and reading at 420 nm in a spectrophotometer.

At the end of the experimental period, rats were anesthetized with sodium pentobarbital (30 mg/kg) and placed on thermoregulated pads. To assess MAP, a catheter was placed in the right femoral artery and connected to a pressure transducer (Model p23 db, Gould, Puerto Rico); then, 5 serial measures were computed every 2 minutes and averaged. Subsequently, a laparotomy was performed to expose and dissect the left renal artery. Finally, an ultrasound probe was placed (1RB, Transonic Systems), and 5 RBF recordings were computed and averaged along with 5 additional MAP measurements. RVR was calculated as RBF divided by MAP.

### Kidney harvesting and histological evaluation.

After MAP and RBF recordings, the right kidney was ligated and removed; then, renal cortex and medulla were manually dissected. Both sections were frozen and stored at –70°C for further analysis. The left kidney was perfused through the femoral artery with 20 mL of 0.9% saline solution and then with 20 mL of 4% paraformaldehyde solution. It was finally embedded in paraffin for histological evaluation. The animals were euthanized using a sodium pentobarbital overdose (100 mg/kg) at the end of the experimental study.

Sections of 3 μm were obtained from paraffin-embedded and stained with periodic acid–Schiff (PAS) or Picrosirius red. Then, high-power fields (HPFs) were digitalized in a blinded fashion using a camera incorporated to a Nikon Light microscope and uploaded to the NIS-Elements software. To evaluate kidney injury at the tenth day, we assessed 10 HPFs (200×) for each PAS-stained kidney cortex using the Khalid tubular score (61). Each HPF was scored as having: no damage = 0; loss of brush border in < 25% of tubular cells = 1; loss of brush border in > 25% of cells with thickening of the basal membrane or tubulorrhexis = 2; inflammation, cast formation, or necrosis in < 60% or tubular area = 3; or necrosis in > 60% of tubular area = 4. An average of the 10 HPFs was obtained for each animal. To evaluate kidney fibrosis at the fifth month, 10 HPFs of Picrosirius red–stained kidneys were digitalized, and red-positive area was quantified with the same software. Similarly, glomerulosclerosis was assessed blindly as percentage of affected glomeruli in 10 HPFs of PAS-stained slices. After assessment, injury scores, glomerulosclerosis, and fibrotic areas were unblinded, assigned to the corresponding group,and compared.

### Electron microscopy and ultrastructural evaluation.

For TEM, slices of kidney cortex were immersed into 2.5% glutaraldehyde dissolved in 0.15 M cacodylate buffer (pH 7.2). Then, the cortex was sectioned in small fragments and fixed in the same solution for 24 hours at 48°C. Resulting fragments were postfixed with 1% OsO_4_, dehydrated in graded ethanol solutions, and embedded in epoxy resin Embed 812 (Electron Microscopy Sciences). Thin sections from 70 to 90 nm were placed on copper grids, contrasted with lead citrate “Reynols” solution and uranyl acetate salts, and examined with a FEI Tecnai G2 Spirit BioTwin transmission electron microscope. At least 10 micrographs per rat were digitalized, and the ultrastructure was subjectively evaluated between the 3 experimental groups.

### mRNA level assessment by semiquantitative PCR.

Bulk, kidney cortex RNA was extracted with the TRIzol method, and 1 μg was retrotranscribed to cDNA (28025-013, Invitrogen). Gene expression was evaluated through qPCR (QuantStudio 5, Applied Biosystems) with the TaqMan Universal PCR Master Mix (4304437, Applied Biosystems). We used verified probes against the genes of interest and eukaryotic 18S rRNA as the housekeeping gene (Supplemental Table 1). Relative expression was calculated through the comparative threshold cycle method (2^–ΔΔCt^).

### Mitochondrial extraction.

For mitochondria isolation, 150–200 mg of cortex were washed with saline 0.9% and homogenized gently on ice using mitochondrial isolation buffer (MIB1x, mannitol 210 mM, sucrose 70 mM, HEPES 5 mM, EGTA 1 mM, fatty acid–free BSA 0.5% [pH 7.2]); then, mitochondrial fraction was obtained through differential centrifugation (800*g* at 4°C for 10 minutes; 8,000*g* at 4°C for 10 minutes) and resuspended in MIB1x or NADH extraction buffer (see below) according to the experimental protocol. For technical considerations, 15 rats were used for the 10-day evaluation (*n* = 5/group); for the 5-day experiments, all rats were used.

### mtNAD^+^/NADH quantification.

The NAD species (NAD^+^ and NADH) were measured in mitochondrial fractions of the renal cortex according to the manufacturer’s instruction (MAK037, Sigma-Aldrich). Briefly, mitochondrial fractions were homogenized in 400 μL of extraction buffer and spun at 10,000*g* at 4°C for 5 minutes in a 10 kDa centrifugal filter device for sample deproteinization, supernatant was recovered in each sample. A volume of 200 µL was heated at 60°C to decompose NAD, leaving only NADH (NADH-only); the rest contained both NAD^+^ and NADH (NADtotal). Samples were transferred to a 96-well plate. To perform the assay, 100 μL of a cycling master mix was added to each well and incubated for 5 minutes, followed by a 1-hour incubation with 10 μL of NADH developer. The plate was read at 450 nm. NAD was calculated as the NAD^total^ – NADH and reported in terms of pmol/μg of mitochondria for each species, as well as the NAD^+^/NADH ratio.

### Protein expression by Western blot.

Kidney cortex samples were homogenized with a lysis buffer containing 50 mM HEPES (H10160, MilliporeSigma), 250 mM NaCl (S988, MilliporeSigma), 5 mM EDTA (1230, Meyer), 0.1% NP-40 (19828, usb) at pH 7.4, and complete protease inhibitor (11697498001, Roche). For mitochondrial fractions, samples obtained after extraction were resuspended in 500 μL of MIB1x. Protein concentration was evaluated by the Lowry Protein Assay (5000113 and 5000114, Bio-Rad). In total, 20–60 μg of protein was loaded to polyacrylamide denaturing gels and electrophoresed. Subsequently, proteins were transferred to polyvinyl difluoride membranes at 18 V for 80–100 minutes. Membranes were blocked for 90 minutes with 5% blotting agent-TBS and incubated with the primary antibody overnight at 4°C. Then, membranes were washed with 0.1% Tween-TBS and incubated with HRP-coupled secondary antibody for 90 minutes. After washing, membranes were placed in the iBright CL1500 Imaging System, and protein expression was assessed through chemiluminescence and digitalized at 300 dpi. For whole cortex lysates, the expression of GAPDH was used as loading control. For mitochondrial isolates, Coomassie blue (CB) staining was used as a loading control, since the typical control proteins used for this matter are affected by IRI (62, 63). Expression was calculated with densitometric analysis of both the gene of interest and GAPDH/CB using the ImageJ Software version 1.51 (NIH). See the used antibodies in Supplemental Table 2.

### IHC for DRP1.

IHC stain was performed using the ultraView Universal DAB detection kit (Ventana-Roche) following the manufacturer’s instructions. Briefly, 4 μm sections were obtained from paraffin-embedded kidney samples; they were fixed on Kling-On charged glass slides (Biocare Medical) and subjected to deparaffinized through xylene and decreasing series of ethanol as per standard procedures. Antigen recuperation was heat induced by immersing slides in Diva Decloaker (Biocare Medical) and boiled for 20 minutes in a pressure cooker. After blocking endogenous peroxidase activity, slides were incubated with primary anti-dynamin related protein-1 antibody (Santa Cruz Biotechnology Inc., sc-271583) at a 1:50 dilution for 60 minutes. Subsequently, they were incubated with the HRP Multimer for 15 minutes, followed by the application of a freshly prepared substrate (3,3′-diaminobenzidine tetrahydrochloride chromogen + H_2_O_2_). The reaction was terminated after 1 minute of incubation on all slides, and counterstaining was performed with hematoxylin. Finally, 10 HPFs per rat were digitalized as described above to assess the location of Drp1 overexpression.

### Immune cell infiltration by flow cytometry.

After homogenization and incubation with collagenase and DNase, 30–50 mg renal cortex biopsies were manually passed through a cell strainer to get a cellular suspension. To eliminate RBC remnants, samples were incubated with Ammonium-Chloride-Potassium lysing buffer (Thermo Fisher Scientific, A10492-01) and were then centrifugated at 300*g* at 4°C for 5 minutes and resuspended on 1 mL PBS FCS 2% (Gibco). Cell suspension was incubated for 1 hour with the mix of the fluorophore-labeled antibodies (Supplemental Table 1). Cell quantification was acquired in a Novocyte Quanteon (Agilent) cytometer, and the gating strategy is shown in Supplemental Figure 1. Data analysis was performed using NovoExpress software (v1.5.6).

### Statistics.

Data distribution of continuous variables was assessed with Shapiro-Wilk test. Homoscedasticity was evaluated with the Bartlett’s test. Log transformation was performed as needed. Comparison of means was performed using ANOVA F-test or Kruskal-Wallis as appropriate. For the comparison of CrCl alone, a 2-way ANOVA was utilized, while all other mean comparisons employed either 1-way ANOVA or the Kruskal-Wallis test. Post hoc testing was performed with Tukey’s or Dunn’s test as appropriate, and with Šidák test for CrCl recovery. To conduct a longitudinal analysis of CrCl, a 2-way ANOVA for repeated measures was employed. For the assessment of proteinuria and body weight, linear mixed modeling was used to evaluate fixed effects, incorporating a by-subject random effect on the intercept, with posterior Bonferroni adjustment to perform pairwise comparisons between means. GraphPad 6 and R version 4.1.2 were used for analysis and figure design. *P* < 0.05 were considered significant.

### Study approval.

All experiments were conducted in accordance with *Guide for the Care and Use of Laboratory Animals* (National Academies Press, 2011) and were approved by the Animal Care and Use Committee of our Institution with the protocol no. CICUAL NMM-2015-20-23-1.

### Data availability.

All data underlying this article are available in the Supporting Data Values file. Additional information may be provided upon request.

Prior publication: The results presented in this paper have only been published previously as an abstract at the International Podocyte Conference 2023 (Philadelphia, Pennsylvania, USA) and at the Kidney Week 2023 (Philadelphia, Pennsylvania, USA).

## Author contributions

NAB and MAMR conceived and design research. MAMR, HB, IGS, JMGR, MERV, and RPV performed animal and molecular experiments. LAVV, JCLC, FR, FC assisted with concrete experimental techniques. MAMR, FR, and NAB analyzed data. MAMR, HB, and NAB interpreted results. MAMR and NAB prepared figures. MAMR and NAB drafted the manuscript. MAMR and NAB revised the article for critical intellectual content. MAMR, HB, IGS, JMGR, MERV, LAVV, JCLC, RPV, FR, FC, and NAB approved the final version of the manuscript.

## Supplementary Material

Supplemental data

Unedited blot and gel images

Supporting data values

## Figures and Tables

**Table 3:**
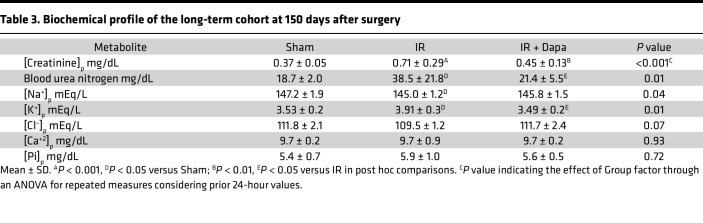
Biochemical profile of the long-term cohort at 150 days after surgery

**Table 2:**
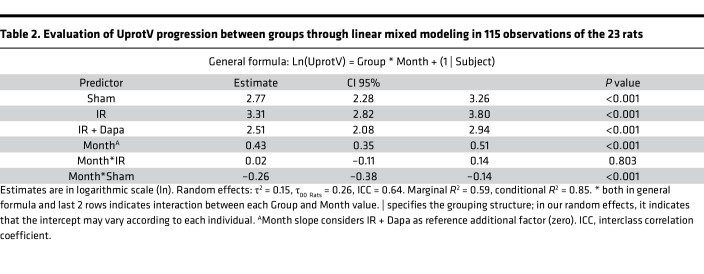
Evaluation of UprotV progression between groups through linear mixed modeling in 115 observations of the 23 rats

**Table 1:**
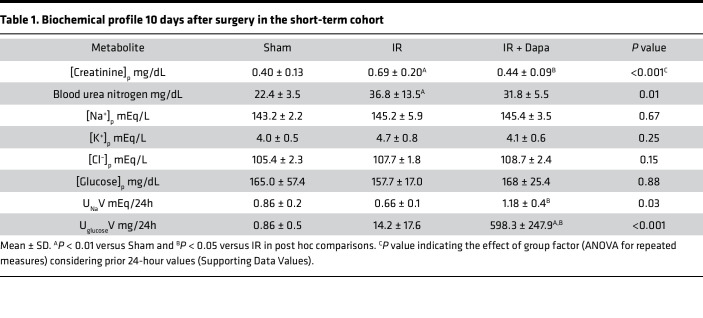
Biochemical profile 10 days after surgery in the short-term cohort

## Version 1 03/22/2024

Electronic publication
