# Supplementary material for: Transient inhibition of sodium-glucose cotransporter 2 after ischemia/reperfusion injury ameliorates chronic kidney disease
Source: JCI Insight. 2024 Mar 22;9(6):e173675. doi: 10.1172/jci.insight.173675 (PMC11063941; doi:10.1172/jci.insight.173675)
Supplement: Supplemental data [file jciinsight-9-173675-s206.pdf]

Transient inhibition of the Sodium-Glucose Cotransporter 2 early after ischemia/reperfusion injury ameliorates Chronic Kidney Disease in rats .

Miguel Ángel Martínez-Rojas[1,2], Hiram Balcázar[1,2], Isaac González-Soria[1,2], Jesús Manuel González-Rivera[1,2], Mauricio E. Rodríguez-Vergara[1,2], Laura A. Velazquez-Villegas[3], Juan Carlos León-Contreras[4], Rosalba Pérez-Villalva[1,2], Francisco Correa[5], Florencia Rosetti[6], and Norma A. Bobadilla[1,2]

Supplemental Figures and Tables:

1. Supplemental Figure 1: Gating strategy used in flow cytometry experiments.
2. Supplemental Figure 2: SGLT2 inhibition with dapagliflozin reduced myeloid infiltration 10 days after AKI.
3. Supplemental Figure 3: Cytokine and inflammatory profile in kidney cortex after 10 days of reperfusion and dapagliflozin administration.
4. Supplemental Table 1: Post hoc multiple comparisons of UprotV values between long-term groups.
5. Supplemental Table 2. Probes and Antibodies used in the experiments

A. T cell population

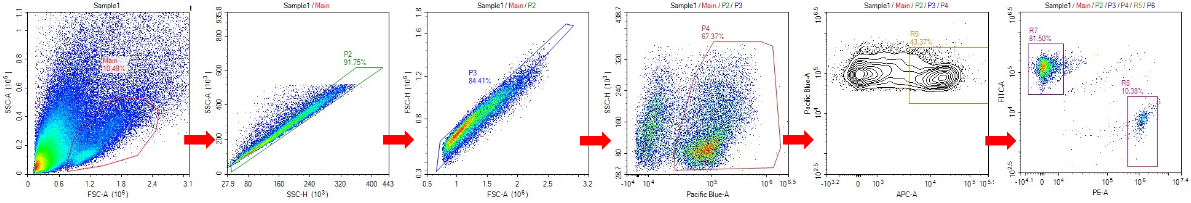

B. Myeloid cell population

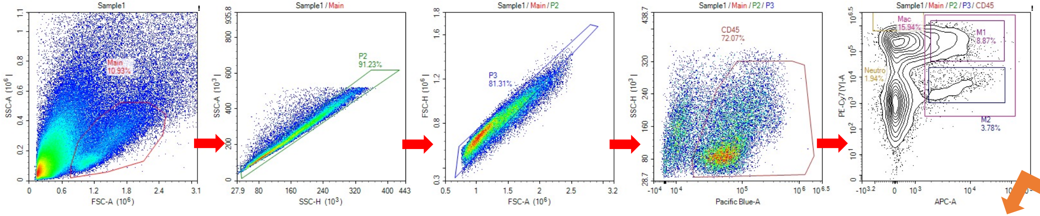

C. Specified fluorophores and antibodies

Stain	T cell-mix	Myeloid-mix
Pacific Blue	CD45	CD45
APC	CD3	CD68
PECy7	-	CD11b
FITC	CD4	CD206
PE	CD8	CD86

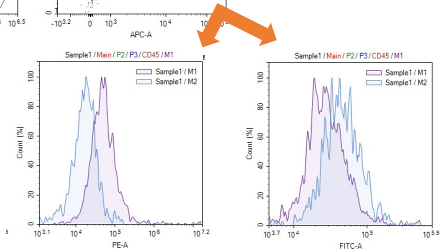

Supplemental Figure 1. Gating strategy used in flow cytometry experiments. All panels show representative dot plots from one IR sample. A. After singlet selection, T cells were identified as CD45$^+$CD3$^+$ cells, and the T helper (CD4$^+$CD8^{neg}) and cytotoxic CD8$^+$CD4^{neg} subpopulations are shown. B. After selection, myeloid cells were identified as CD45$^+$CD11b$^+$ cells, neutrophils were considered as the CD11b^{high}CD68^{neg} population, while macrophages as CD11b$^+$CD68$^+$ cells. From the macrophage populations, two subpopulations were identified: CD11b^{high} and CD11b^{low}, respectively; these cells showed different expression of CD86 and CD206, suggestive of M1- and M2-like phenotypes, respectively. C. The table indicates the antibodies and fluorophore used in the staining.

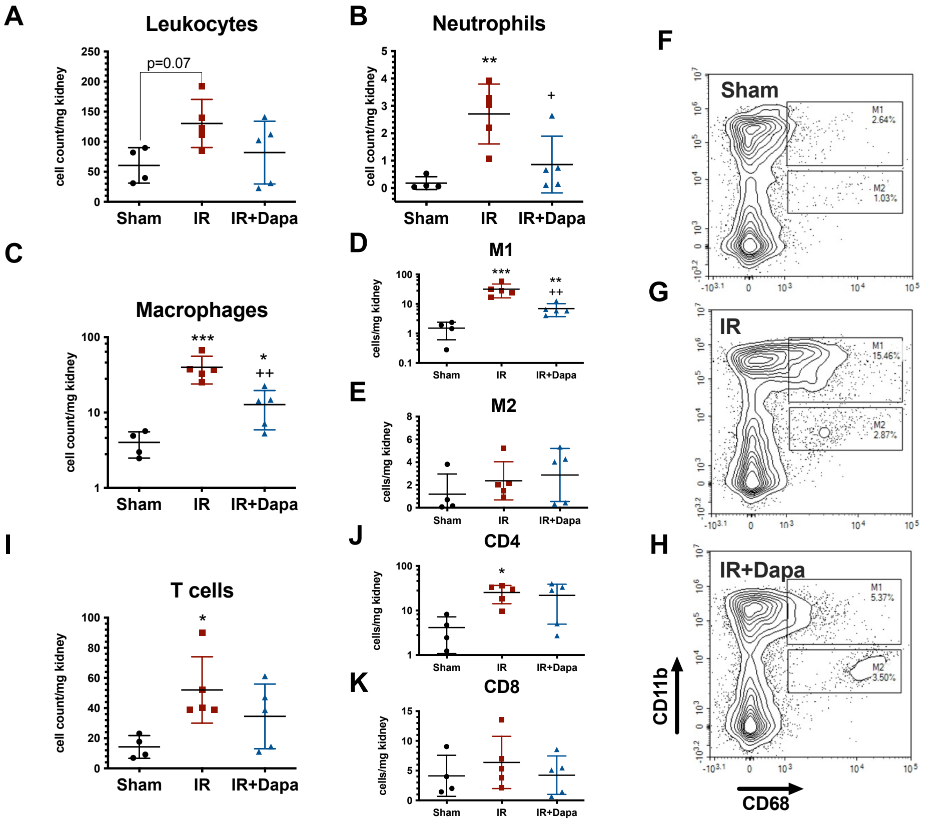

Supplemental Figure 2. SGLT2 inhibition with dapagliflozin reduced myeloid infiltration 10 days after AKI. Flow cytometry analysis of cell infiltrates from renal cortex in the three studied groups: Sham (black circles), IR (red squares), IR+Dapa (blue triangles). A. Total leukocyte number per mg of tissue is shown, B. Total neutrophils per mg, C-E. Total macrophages and their subpopulations, M1-like and M2-like cells, as indicated. F-H. Representative density plots of macrophages populations are shown (gated on live $CD45^{+}$ cells as indicated in Supp Fig 1). I-K. Total T lymphocytes and their subtypes per mg of tissue are presented. Each symbol represents a rat, and the results presented are cumulative from three independent experiments. Statistical differences were analyzed by ANOVA F-test for all panels (Mean ± SD). Data in panels C, D, and J were log-transformed for analysis. *p<0.05, **p<0.01, ***p<0.001 vs Sham; $^{+}$p<0.05, $^{++}$p<0.01 vs IR.

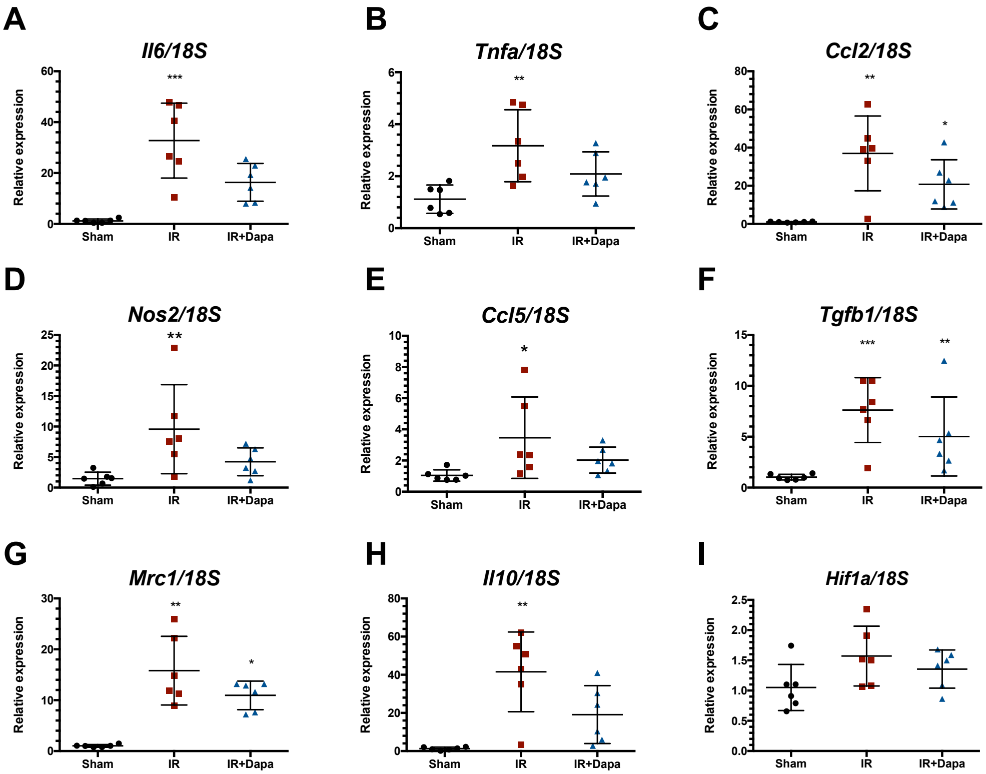

Supplemental Figure 3. Cytokine and inflammatory profile in kidney cortex after 10 days of reperfusion and dapagliflozin administration. Relative mRNA expression of acute-phase molecules A) *Il6,* B) *Tnfa*, C) *Ccl2*, D) *Nos2*, and E) *Ccl5.* Relative expression of profibrotic repair-phase cytokines F) *Tgfb,* G) *Mrc1,* H) *Il10.* I) Also, the expression of *Hif1a* follows a similar trend than inflammatory genes. n= 6 per group. Mean±SD. *p<0.05, **p<0.01, ***p<0.001 vs Sham.

Supplemental Table 1. Post hoc multiple comparisons of UprotV values between long-term groups.

Month	.y.	group1	group2	n1	n2	p	p.signif	p.adj	p.adj.signif
1	logProt	IR+Dapa	IR	9	7	0.0113	*	0.0338	*
1	logProt	IR+Dapa	Sham	9	7	0.464	ns	1	ns
1	logProt	IR	Sham	7	7	0.0681	ns	0.204	ns
2	logProt	IR+Dapa	IR	9	7	0.0053	**	0.0159	*
2	logProt	IR+Dapa	Sham	9	7	0.231	ns	0.693	ns
2	logProt	IR	Sham	7	7	0.00054	***	0.00162	**
3	logProt	IR+Dapa	IR	9	7	0.0277	*	0.0832	ns
3	logProt	IR+Dapa	Sham	9	7	0.0866	ns	0.26	ns
3	logProt	IR	Sham	7	7	0.000815	***	0.00244	**
4	logProt	IR+Dapa	IR	9	7	0.0541	ns	0.162	ns
4	logProt	IR+Dapa	Sham	9	7	0.00432	**	0.013	*
4	logProt	IR	Sham	7	7	0.000075	****	0.000225	***
5	logProt	IR+Dapa	IR	9	7	0.00724	**	0.0217	*
5	logProt	IR+Dapa	Sham	9	7	0.0421	*	0.126	ns
5	logProt	IR	Sham	7	7	0.0000934	****	0.00028	***

Supplemental Table 2. Probes and Antibodies used in the experiments

Target Gene	Reagent	Catalog Number
Eurkaryotic 18S rRNA	Probe	Rn03928990_g1
Interleukin-6 (*Il6*)	Probe	Rn01410330_m1
Tumor Necrosis Factor α (*Tnf*)	Probe	Rn99999017_m1
Chemokine CC motif Ligand 2 (*Ccl2*)	Probe	Rn00580555
Interleukin-10 (*Il10*)	Probe	Rn99999012_m1
Transforming Growth Factor β-1 (*Tgfb1*)	Probe	Rn00572010_m1
Mannose Receptor C Type 1 (*Mrc1*)	Probe	Rn01487342_m1
Kidney Injury Molecule 1 (*Havcr1*)	Probe	Rn00597703_m1
Serpin A3c (*Serpina3c*)	Probe	Rn04289570_m1
Hypoxia-inducible factor 1a (*Hif1a*)	Probe	Rn00577560_m1
Nitric oxide synthase 2 (*Nos2*)	Probe	Rn00561646_m1
Nitric oxide synthase 3 (*Nos3*)	Probe	Rn02132634_s1
Sirtuin 3 (SIRT3)	Mouse Antibody	sc-365175
Mitofusin 1 and 2 (MFN1, MFN2)	Mouse Antibody	ab57602
Dynamin-related protein 1 (DRP1)	Mouse Antibody	sc-271583
Optic Atrophy 1 (OPA1)	Mouse Antibody	sc-393296
OXPHOS	Mouse Antibody	ab110413
Glyceraldehyde 3-phosphate dehydrogenase (GAPDH)	Rabbit Antibody	ab181602
Angiotensinogen (AGT)	Rabbit Antibody	ab213705
PTEN-induced kinase 1 (PINK1)	Rabbit Antibody	P0076
Parkin	Mouse Antibody	P6248
B cell lymphoma 2 (BCL2)	Rabbit Antibody	SAB5701336
Bcl2-Associated X (BAX)	Rabbit Antibody	SAB5701333
Bcl2-induced protein 3 (BNIP3)	Mouse Antibody	sc-56167
NLR family pyrin domain containing	Rabbit Antibody	15101S

3 (NLRP3)		
Antimouse IgG-peroxidase	Goat Antibody	ab6789
Antirabbit IgG-peroxidase	Goat Antibody	A0545
Antirabbit IgG-peroxidase	Mouse Antibody	211-032-171
Antimouse IgG-peroxidase	Goat Antibody	115-035-174
Pacific blue-CD45	F-Antibody*	202225
PE/Cy7-CD11b	F-Antibody	201817
APC-CD68	F-Antibody	130-103-364
PE-CD86	F-Antibody	200307
FITC-CD206	F-Antibody	GTX43682
APC-CD3	F-Antibody	201413
FITC-CD4	F-Antibody	201505
PE-CD8a	F-Antibody	200607

*F-Antibody: Fluorophore-coupled antibody
