# Supplementary material for: Transient inhibition of sodium-glucose cotransporter 2 after ischemia/reperfusion injury ameliorates chronic kidney disease
Source: JCI Insight. 2024 Mar 22;9(6):e173675. doi: 10.1172/jci.insight.173675 (PMC11063941; doi:10.1172/jci.insight.173675)
Supplement: Unedited blot and gel images [file jciinsight-9-173675-s207.pdf]

Transient inhibition of the Sodium-Glucose Cotransporter 2 early after ischemia/reperfusion injury ameliorates Chronic Kidney Disease in rats .

Miguel Ángel Martínez-Rojas[1,2], Hiram Balcázar[1,2], Isaac González-Soria[1,2], Jesús Manuel González-Rivera[1,2], Mauricio E. Rodríguez-Vergara[1,2], Laura A. Velazquez-Villegas[3], Juan Carlos León-Contreras[4], Rosalba Pérez-Villalva[1,2], Francisco Correa[5], Florencia Rosetti[6], and Norma A. Bobadilla[1,2]

Supplemental Western Blot Images
Obtained with the iBright CL1500 Imaging System

Full unedited gel for Figure 2: AGT
30 ug of kidney cortex homogenates

Primary Rabbit ab213705 Antibody: 1:5000
Anti-Rabbit 1:20,000
Exp 5 seg

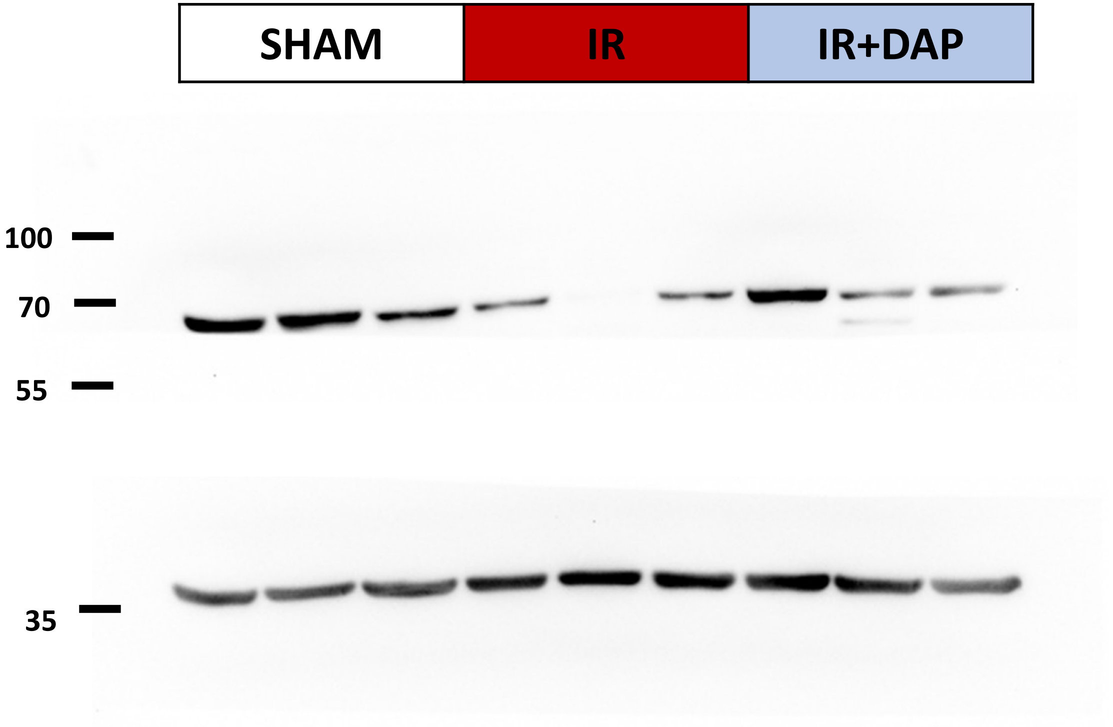

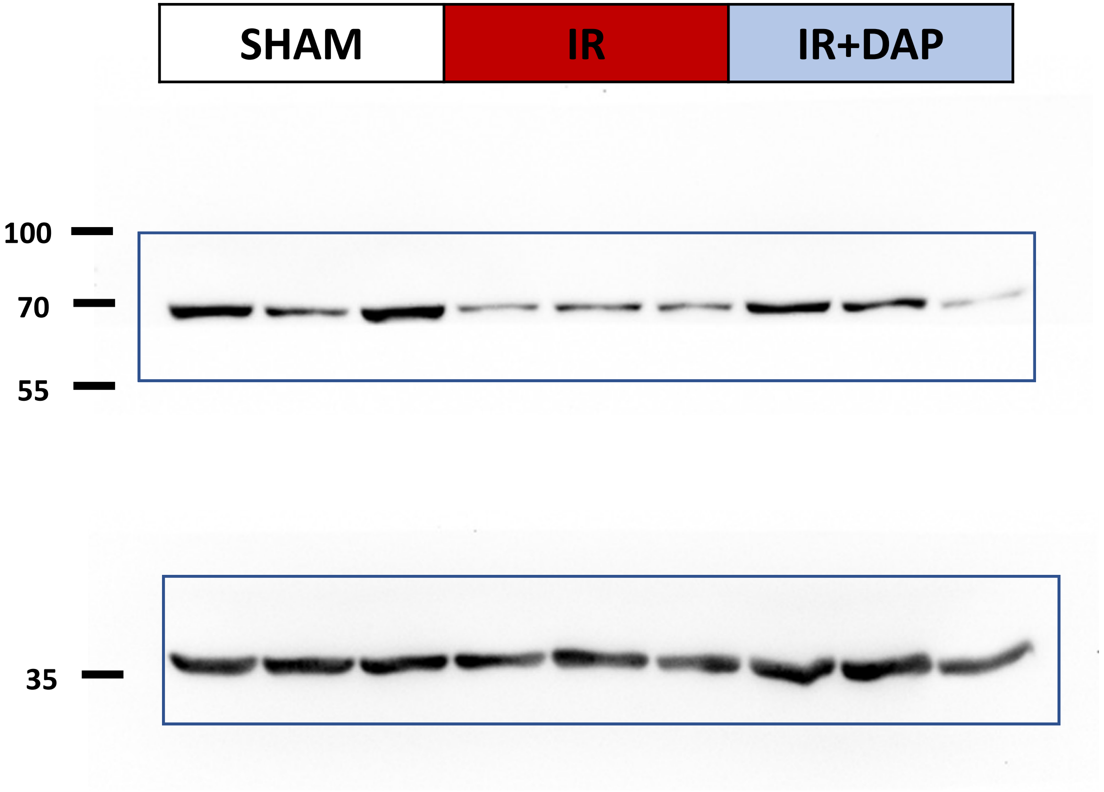

Primary Rabbit GAPDH Antibody: ab181602
1:120,000 Rabbit 1:10,000 Exp 5 s

19/04/23

Full unedited gels for Figure 4 and 7: Mitofusin 1 and 2
20 ug of kidney cortex mitochondria

Primary Mouse Anti-Mfn: 1:2000
Anti-Mouse ab6789 1:40,000 Exp 10 seg

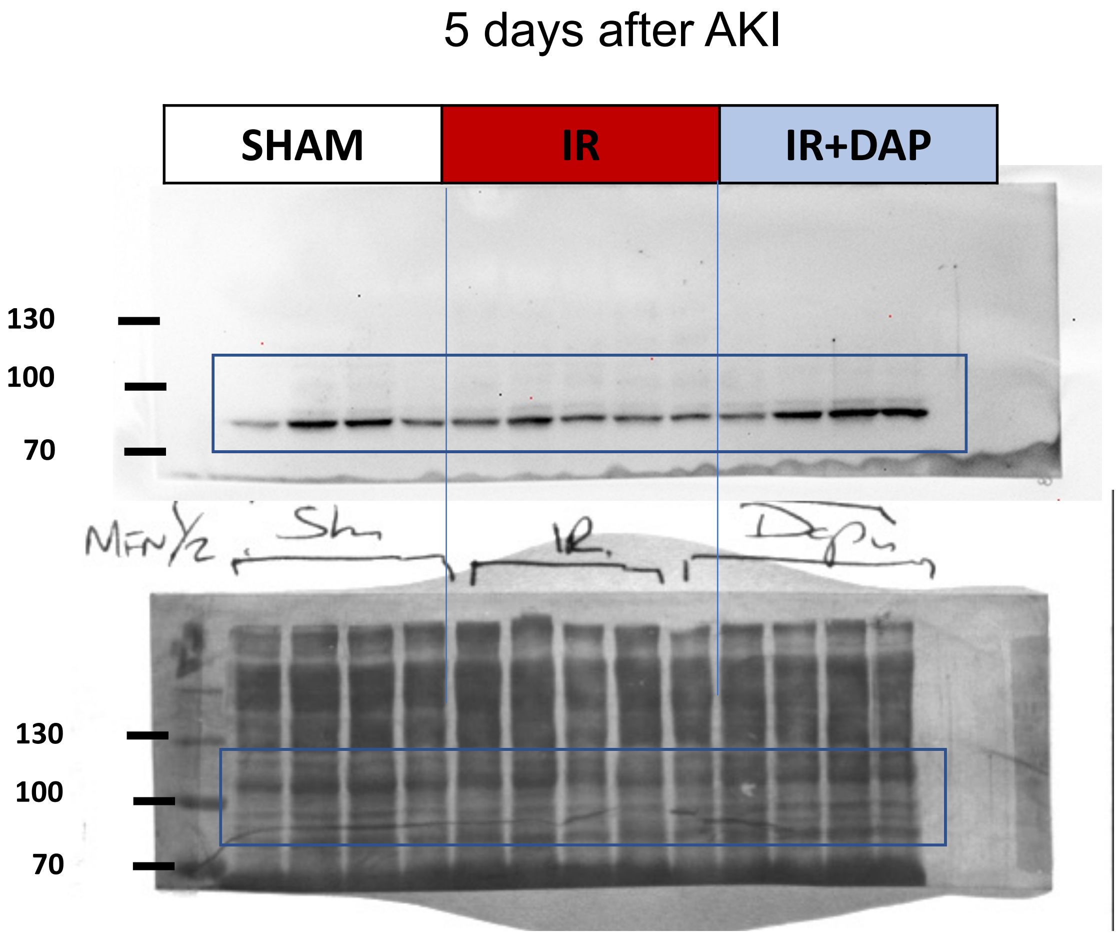

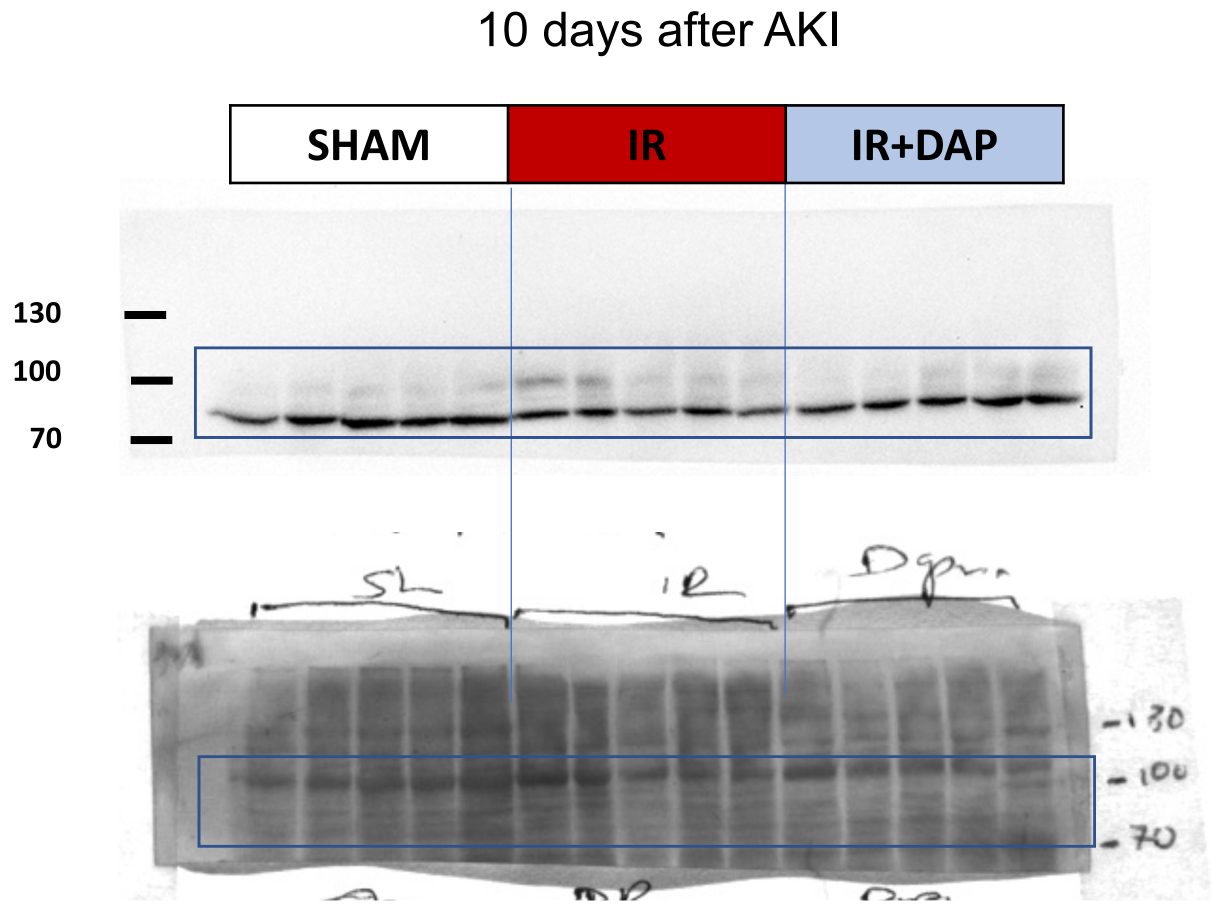

29/11/2023

04/01/2024

Full unedited gel for Figure 4 and 7: Drp1
60 ug of kidney cortex isolated mitochondria

Primary Mouse Anti-Drp1 sc-271583 : 1:500
Anti-Mouse ab6789 1:40,000 Exp 1 min

5 days after AKI

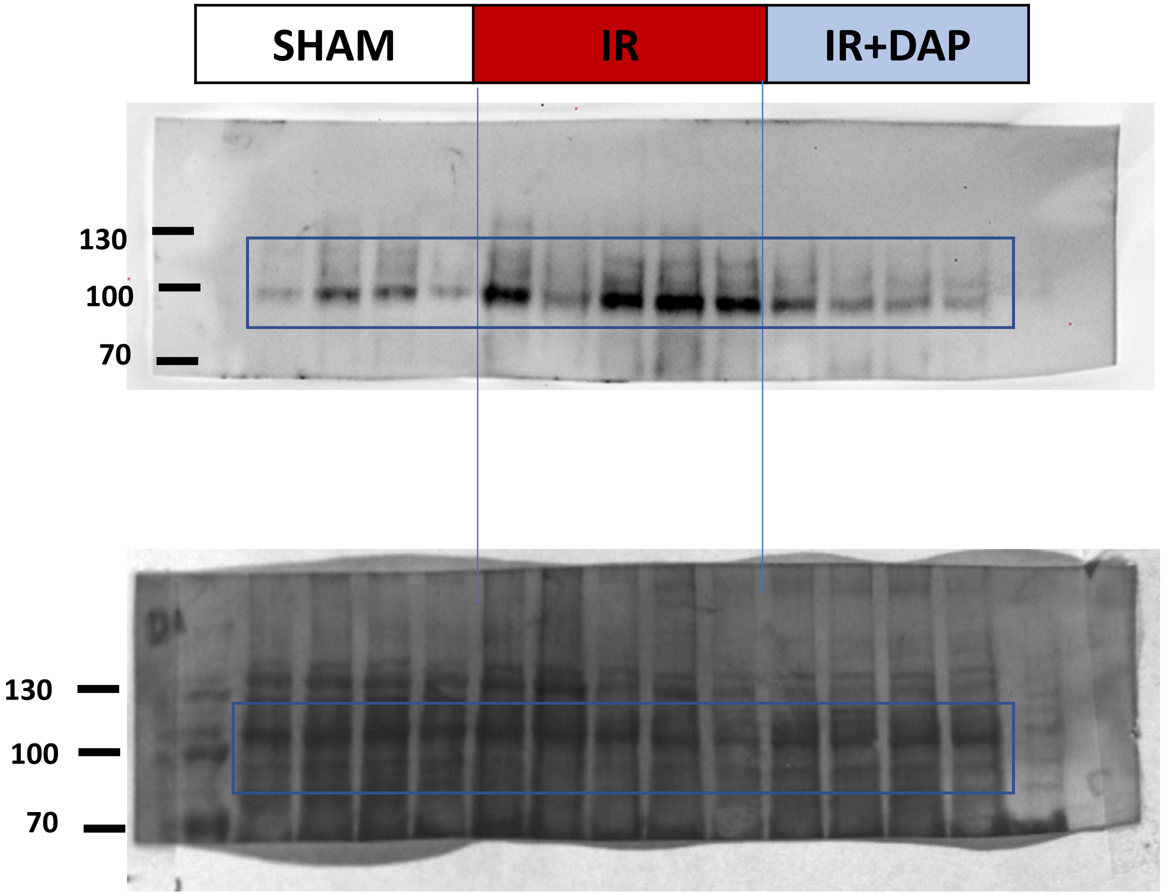

15/12/2023

10 days after AKI

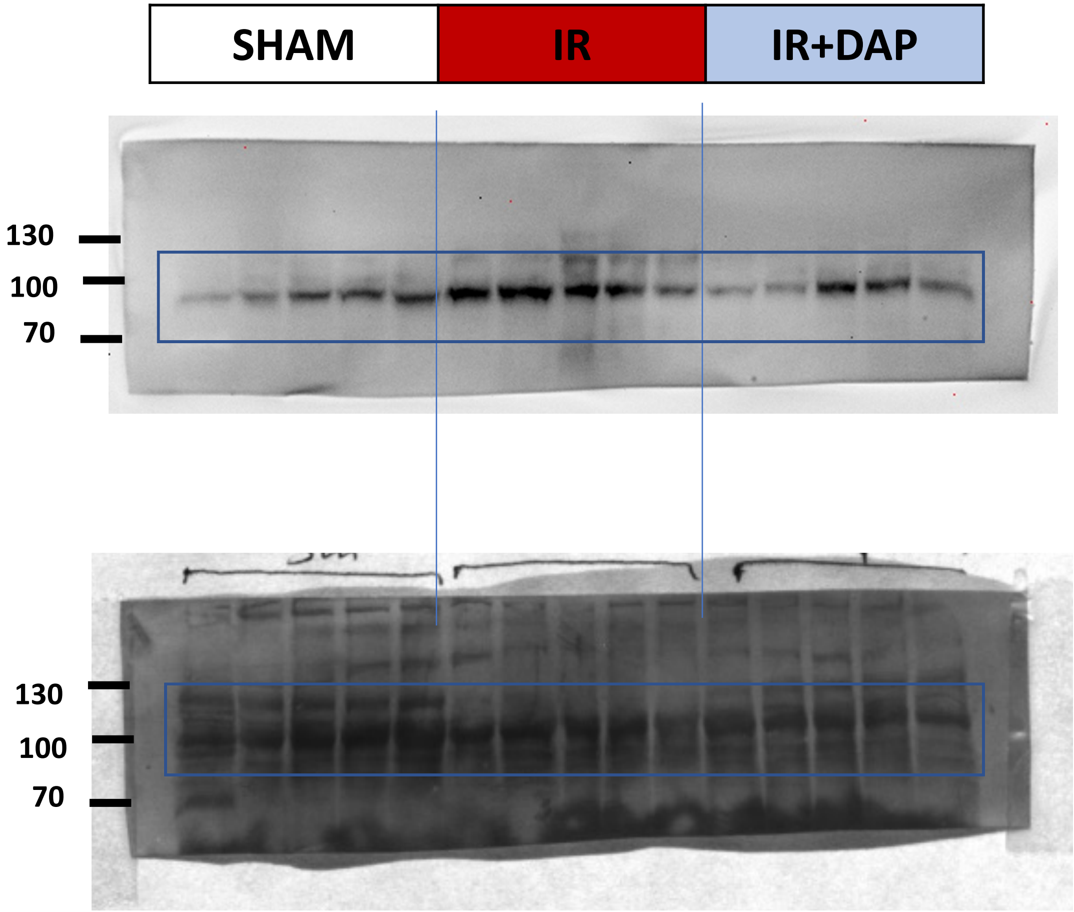

21/12/23

Full unedited gel for Figure 4 and 7: OPA1
60 ug of kidney cortex isolated mitochondria

Primary Mouse Anti-OPA1 sc-393296: 1:500
Anti-Mouse ab6789 1:40,000 Exp 30 seg

5 days after AKI

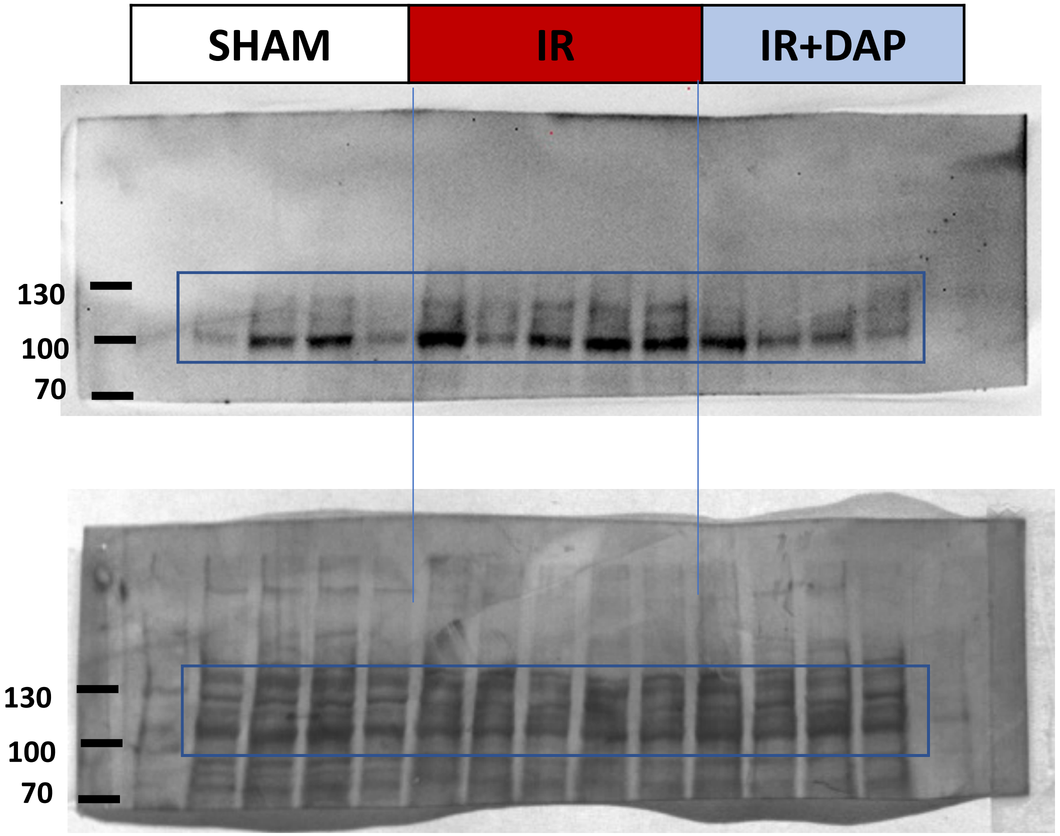

15/12/2023

10 days after AKI

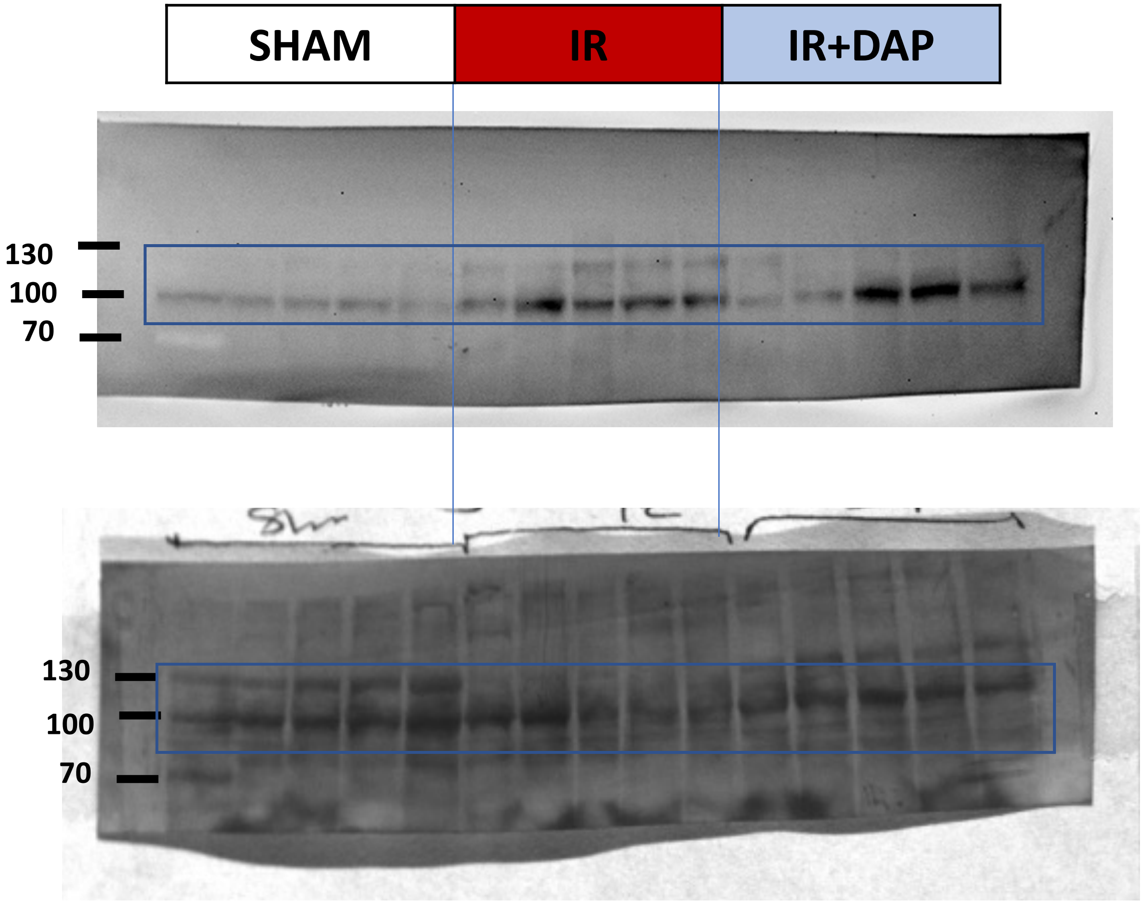

21/12/23

Full unedited gel for Figure 4 and 7: PINK1
20 ug of kidney cortex isolated mitochondria

Primary Rabbit Anti-PINK1 P0076 1:1000
Anti-Rabbit 211-032-171 1:15,000 Exp 10 seg

5 days after AKI

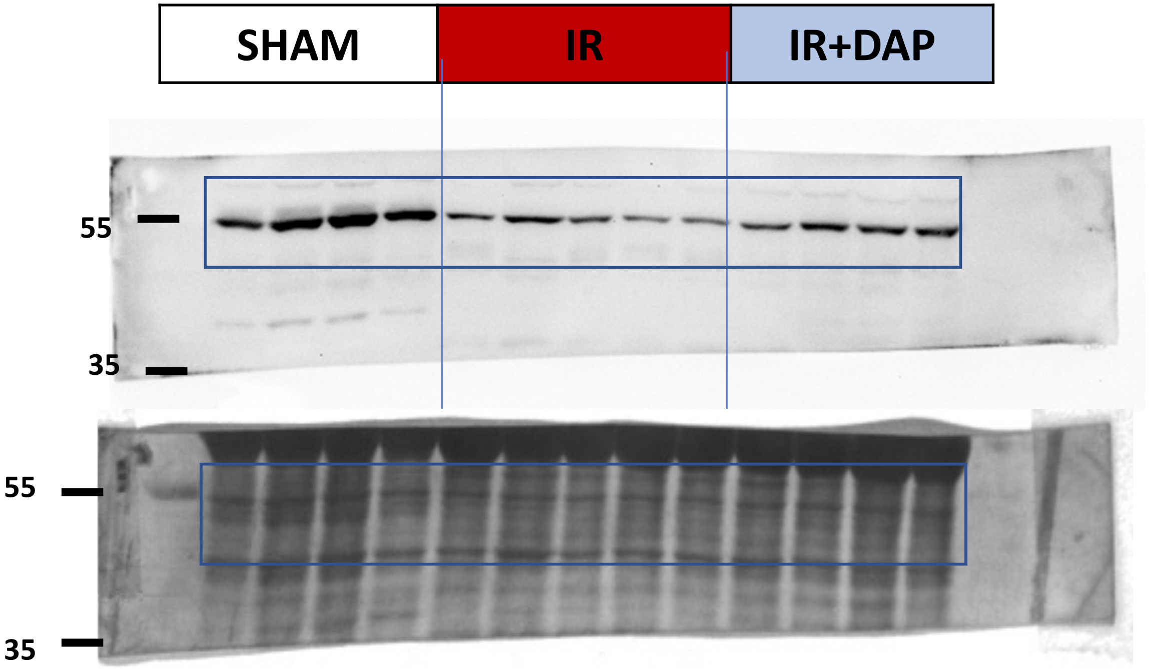

10 days after AKI

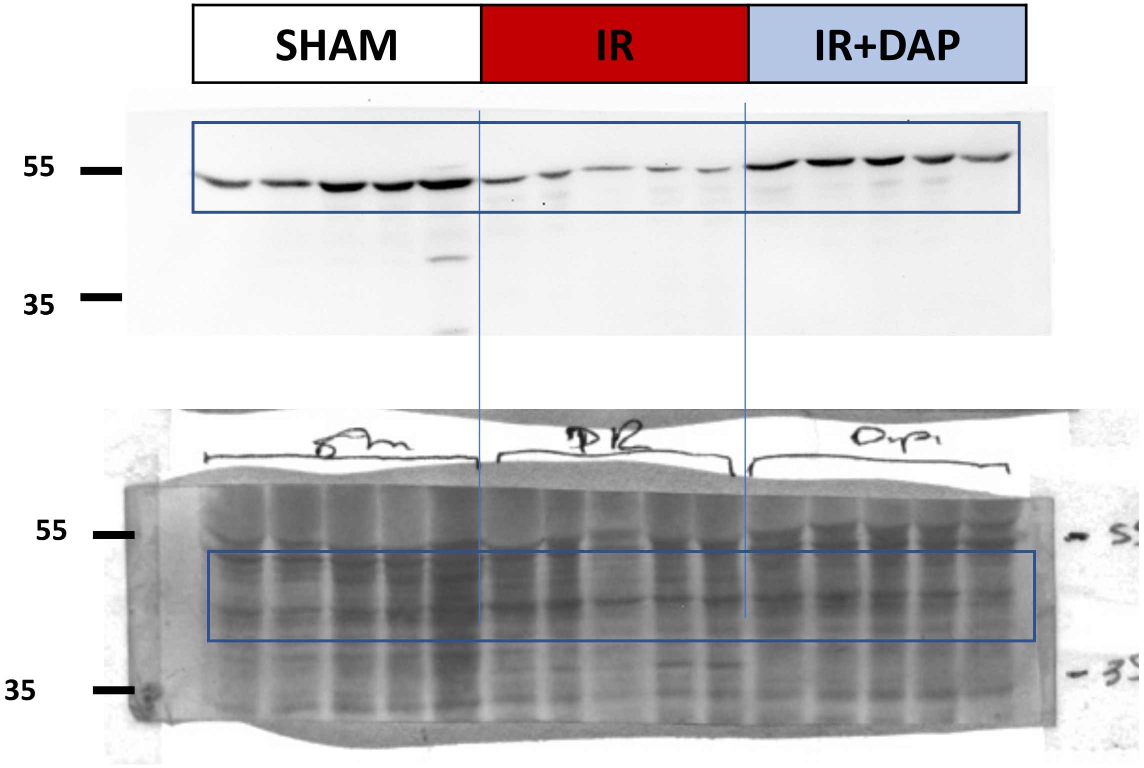

05/12/2023

Full unedited gel for Figure 4 and 7: Parkin

60 ug of kidney cortex isolated mitochondria

Primary Rabbit Anti-Parkin P6248 1:1000
Anti-Mouse ab6789 1:40,000 Exp 3 min

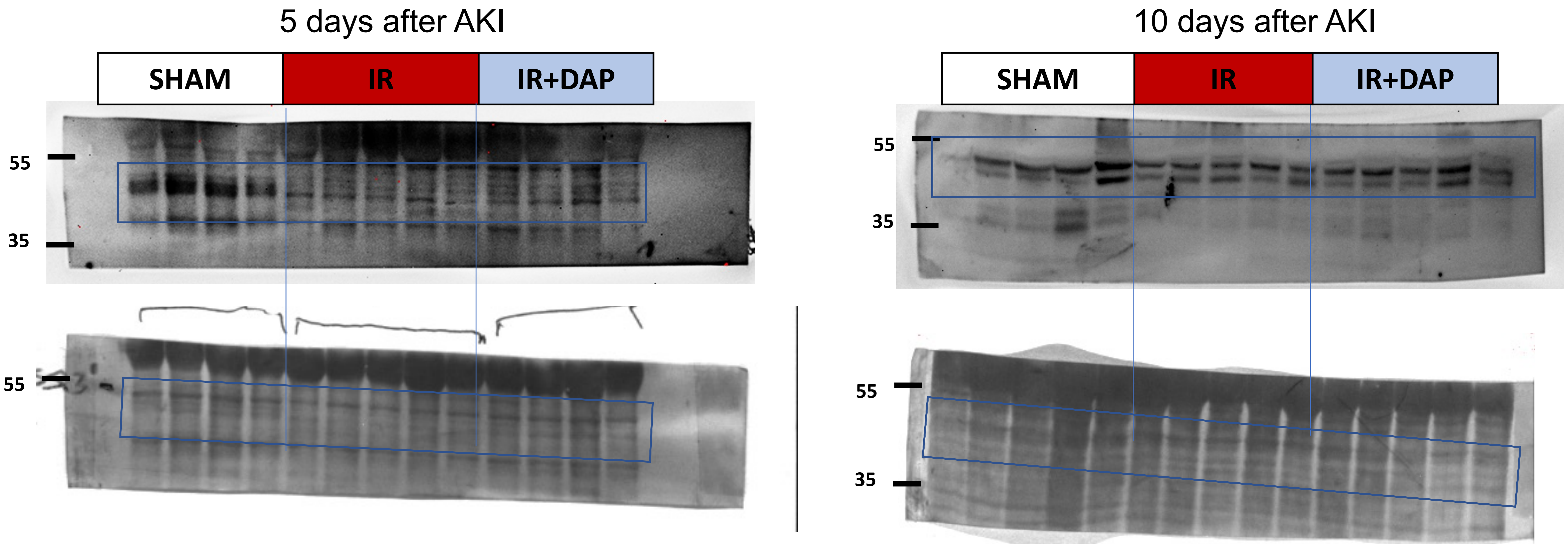

11/12/2023

15/12/2023

Full unedited gel for Figure 5 and 8: Sirt3
30 ug of kidney cortex isolated mitochondria
Primary Mouse Anti-Sirt3 sc-365175 1:1000
Anti-Mouse ab6789 1:40,000 Exp 1 min

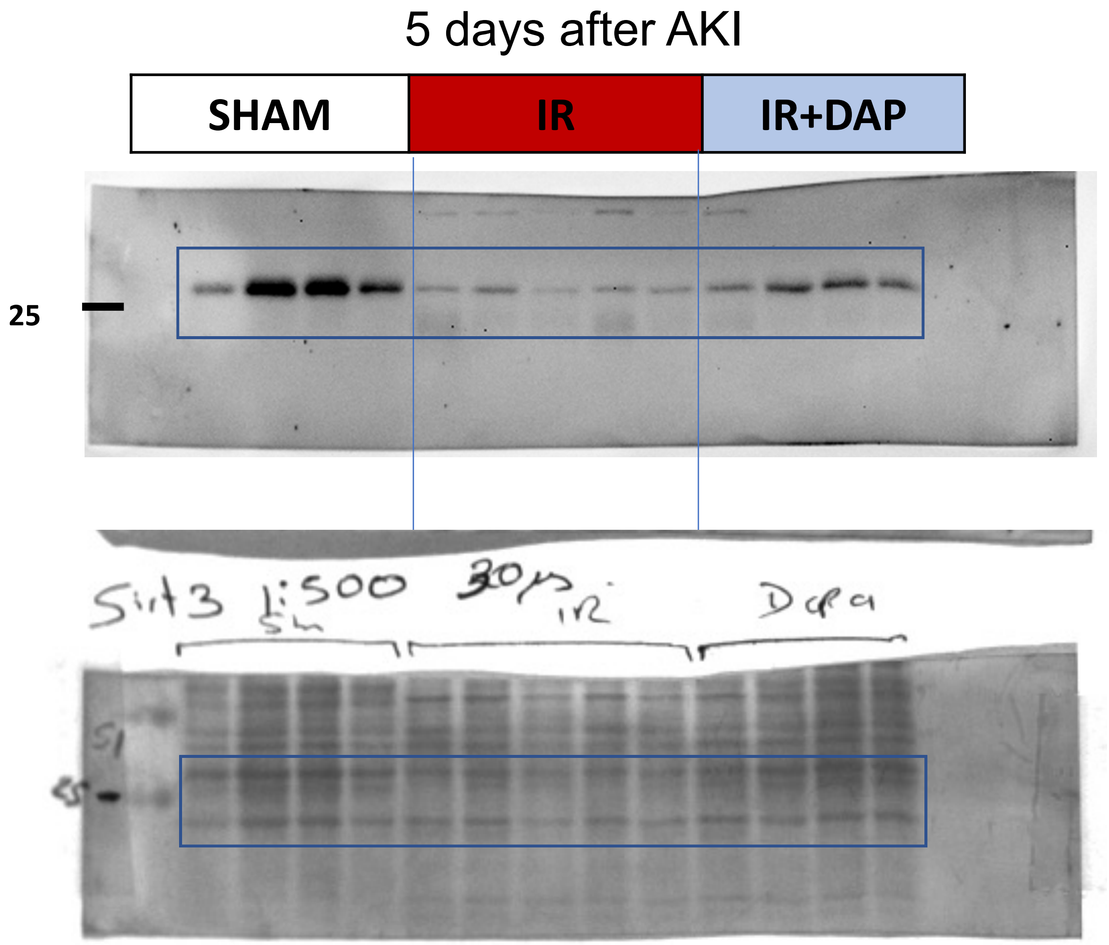

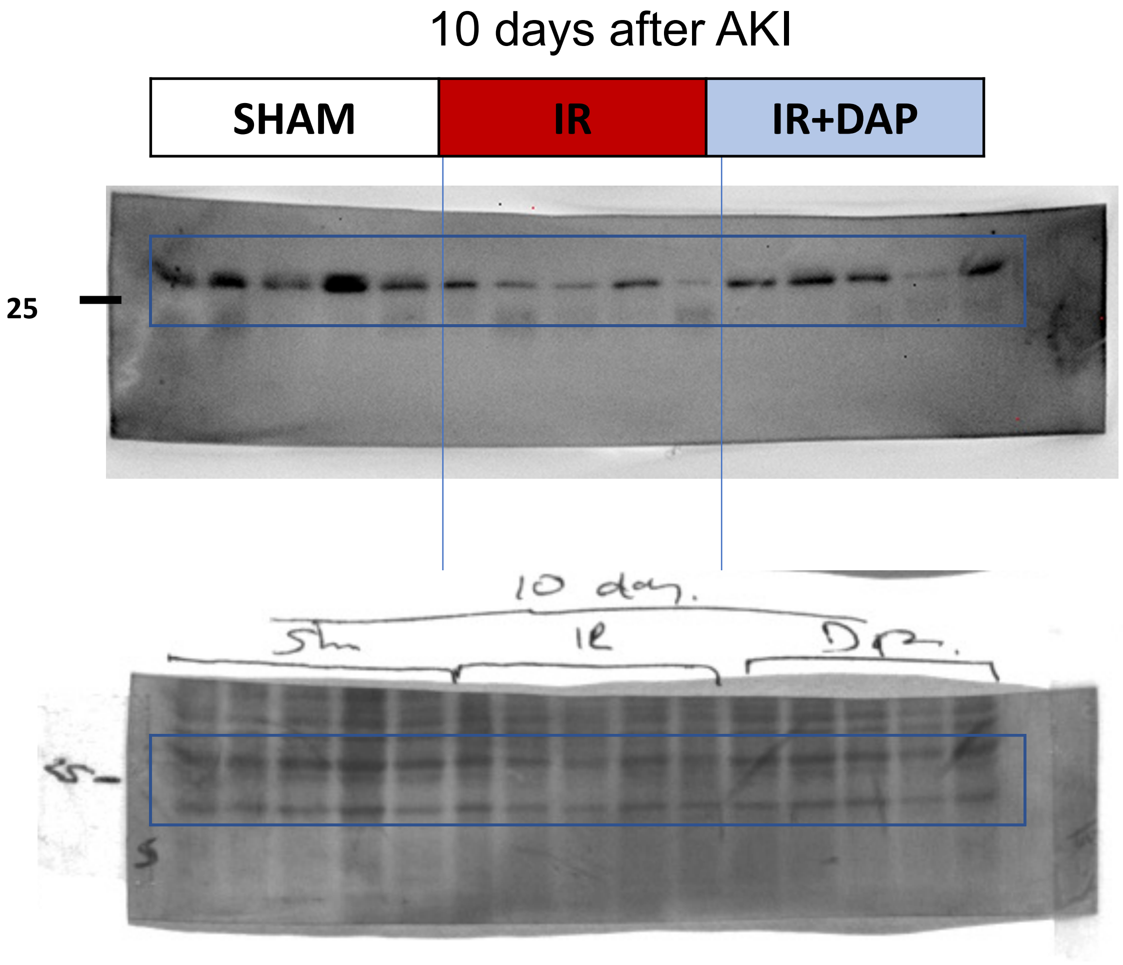

20/12/2023

Full unedited gel for Figure 5 and 8: OXPHOS Complex V, III, I

20 ug of kidney cortex mitochondria

Primary Mouse Anti-OXPHOS ab110413: 1:20,000

Anti-Mouse ab6789 1:40,000 Exp 1 min

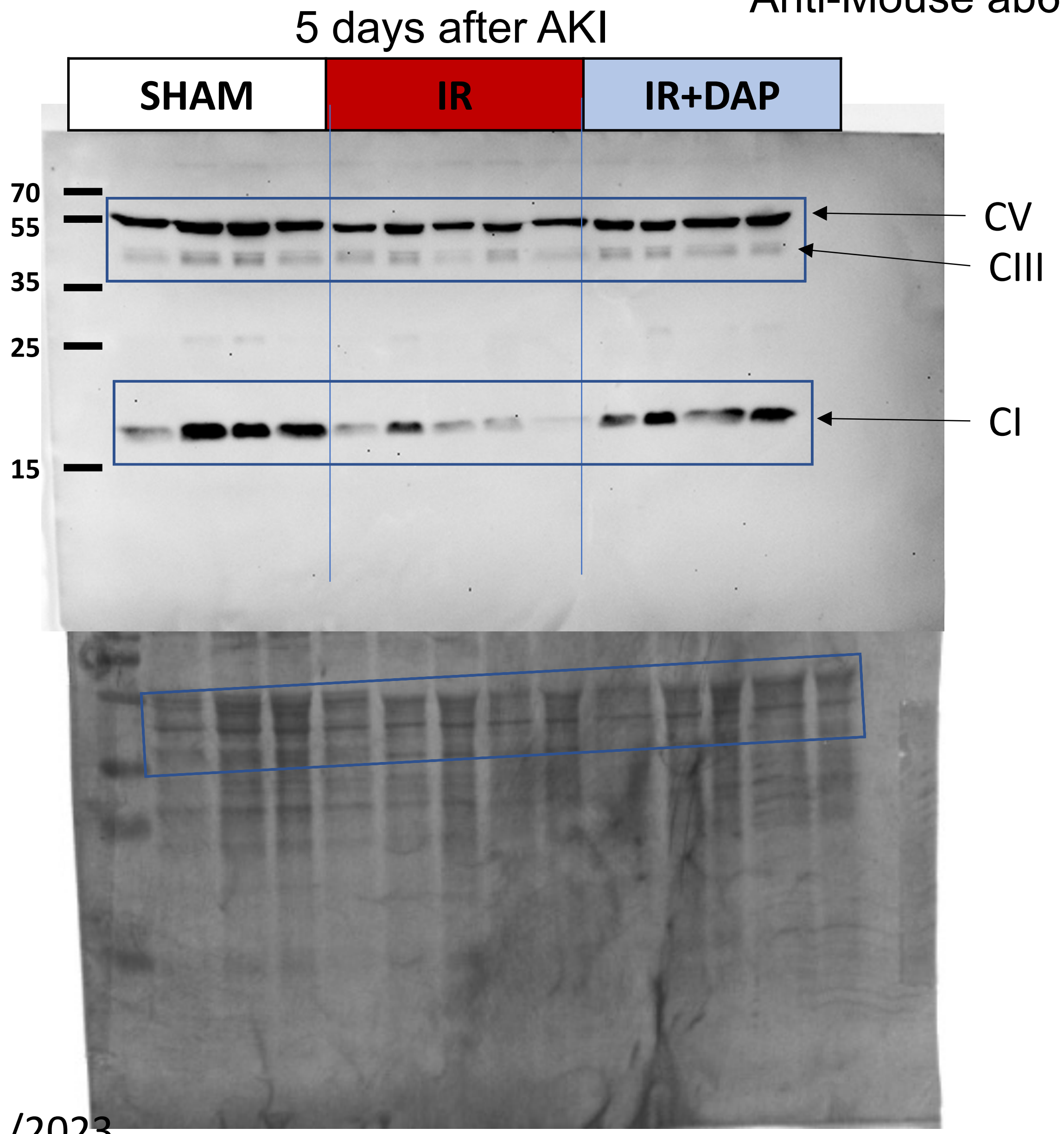

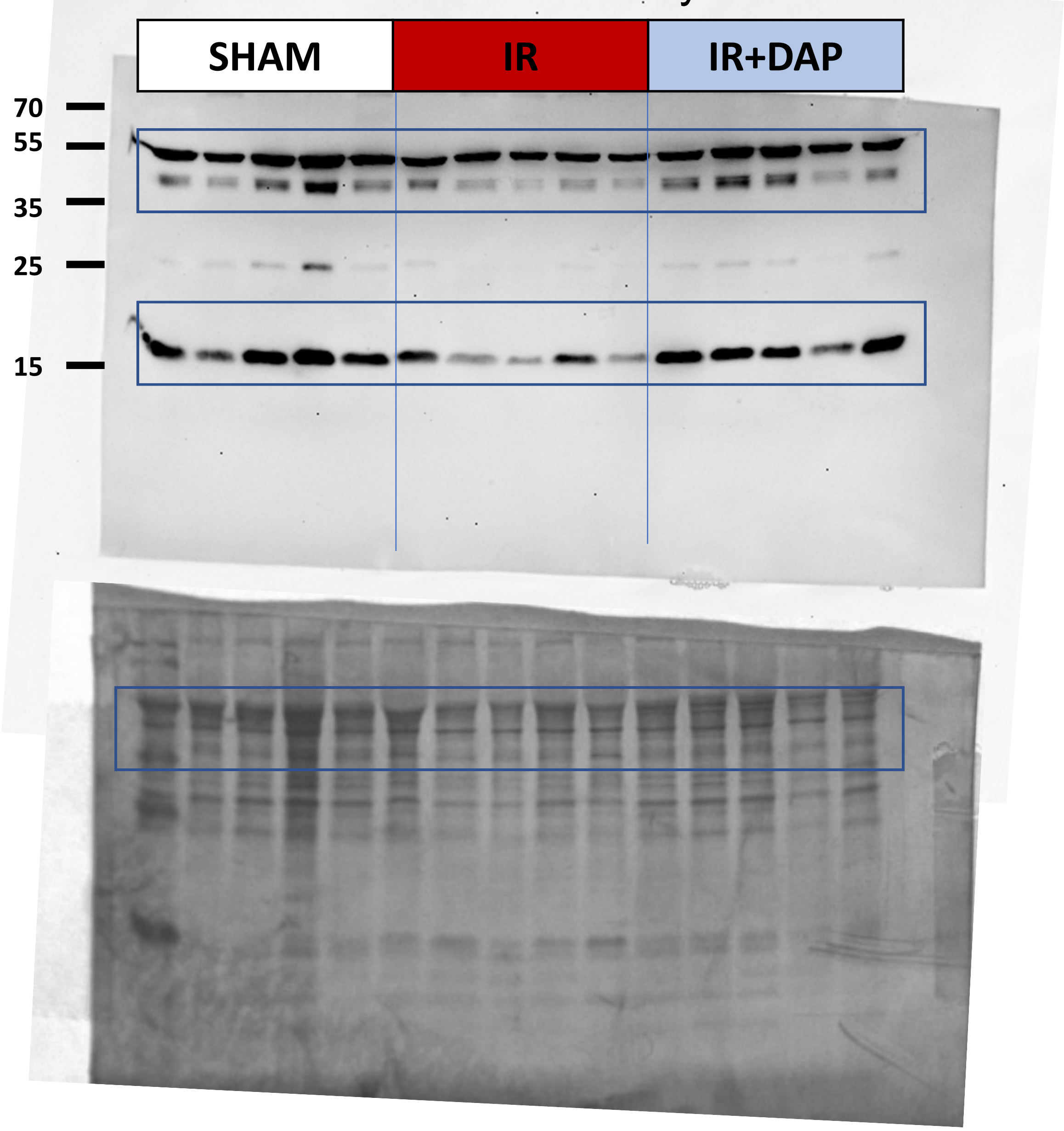

30/11/2023

Full unedited gel for Figure 5 and 8: OXPHOS IV Y II
20 ug of kidney cortex isolated mitochondria

Primary Mouse Anti-OXPHOS ab110413 : 1:20,000
Anti-Mouse ab6789 1:40,000 Exp 1 min

5 days after AKI

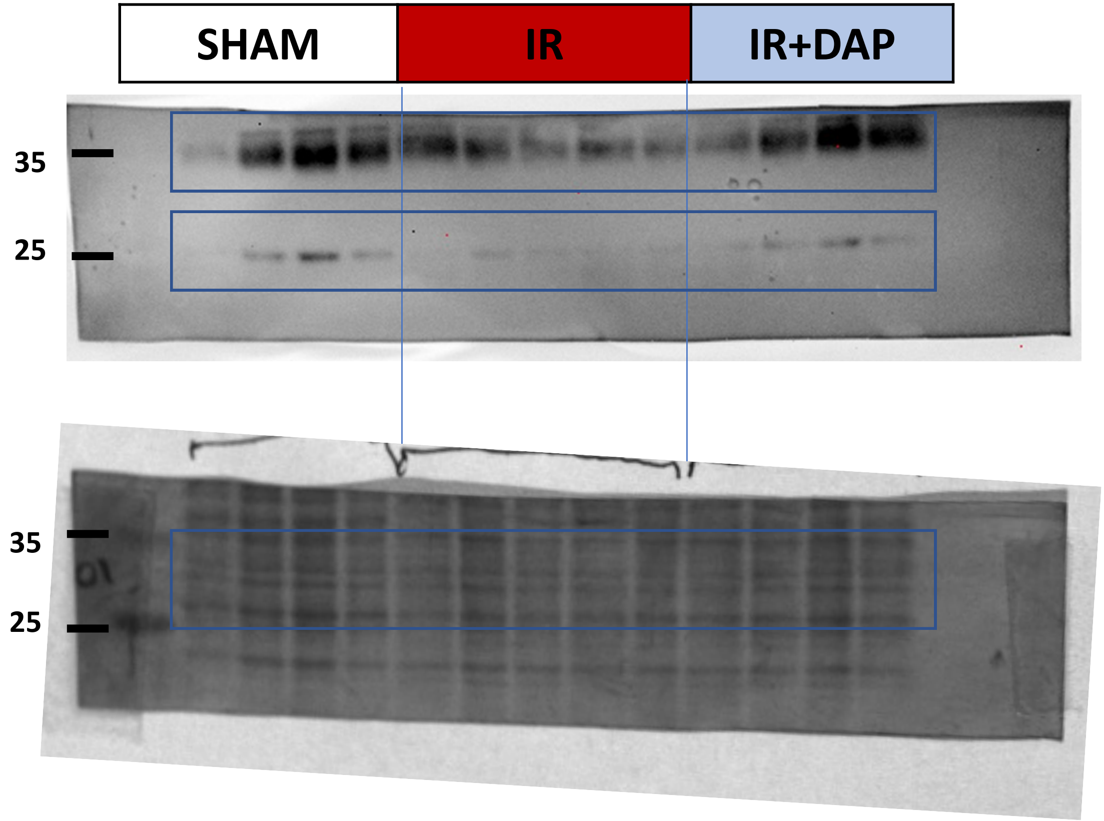

10 days after AKI

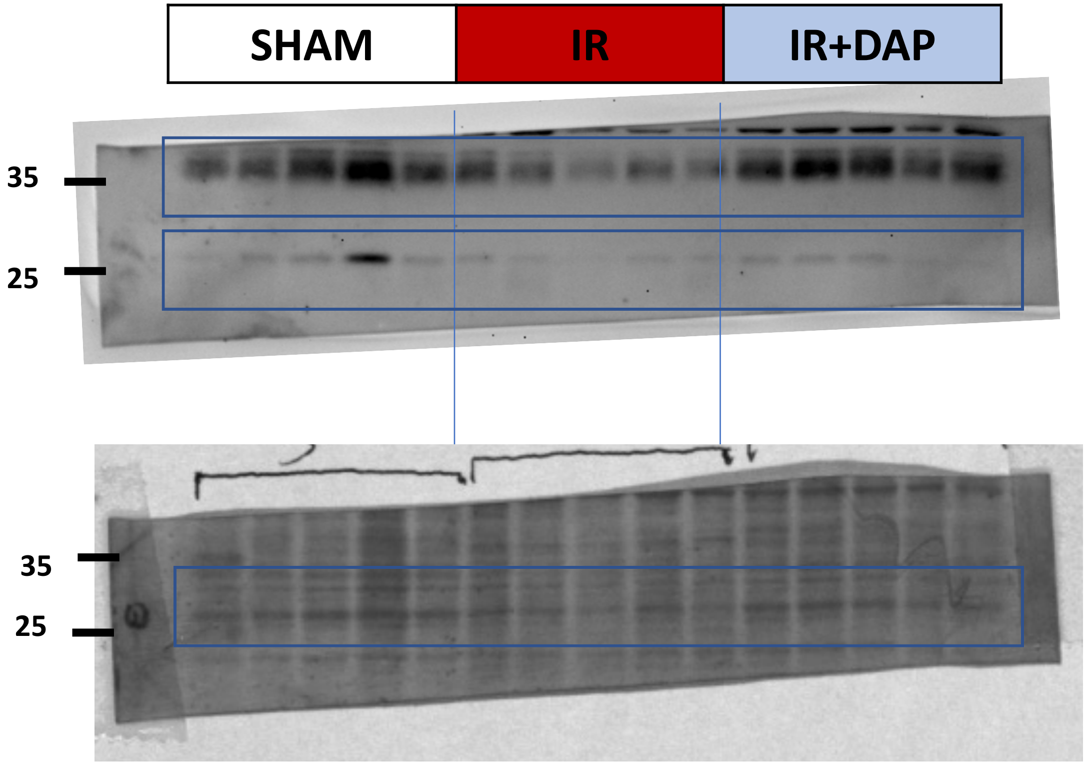

13/12/2023

Full unedited gel for Figure 6 and 8: Bcl2
60 ug of kidney cortex isolated mitochondria

Primary Rabbit Anti-Bcl2 SAB5701336: 1:500
Anti-Rabbit A0545 1:7,500 Exp 1 min

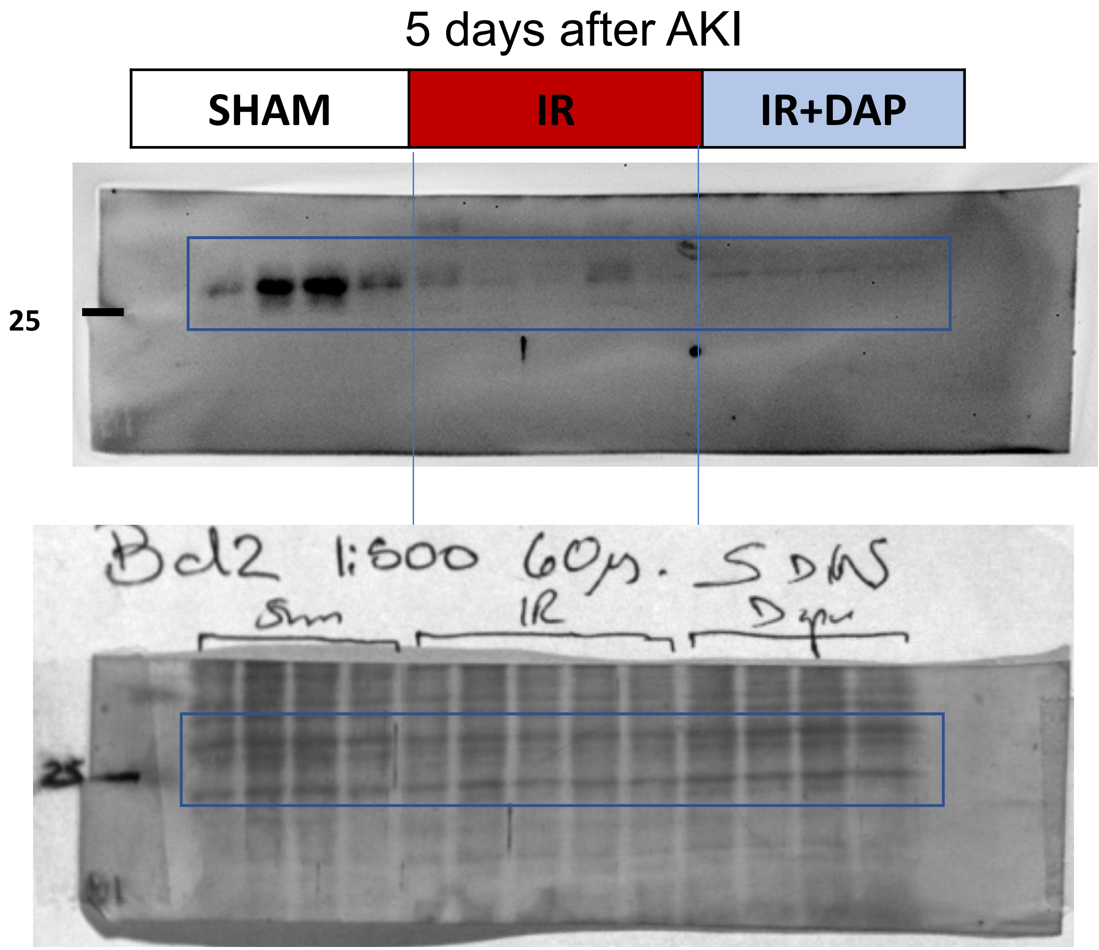

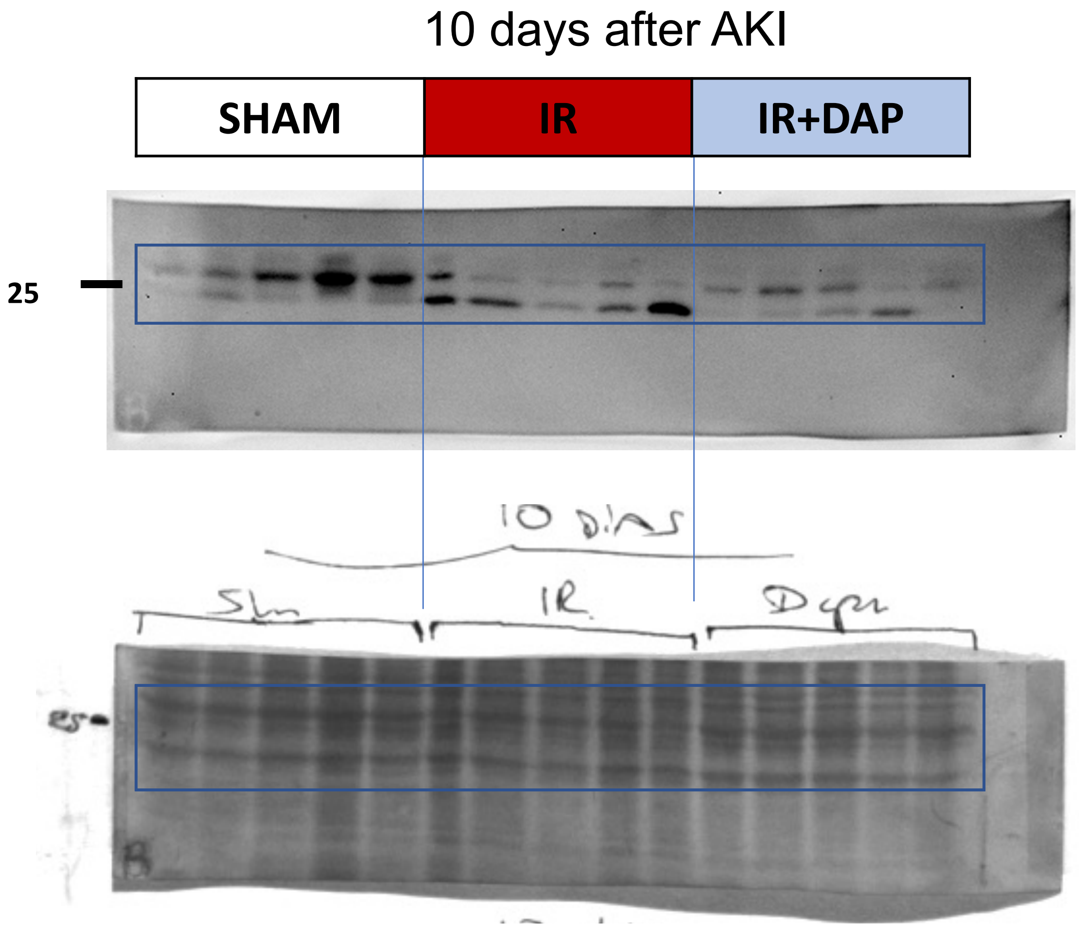

20/12/2023

Full unedited gel for Figure 6 and 8: BAX
20 ug of kidney cortex isolated mitochondria

Primary Rabbit Anti-Bax SAB5701333 : 1:2500
Anti-Rabbit A0545 1:10,000 Exp 30 seg

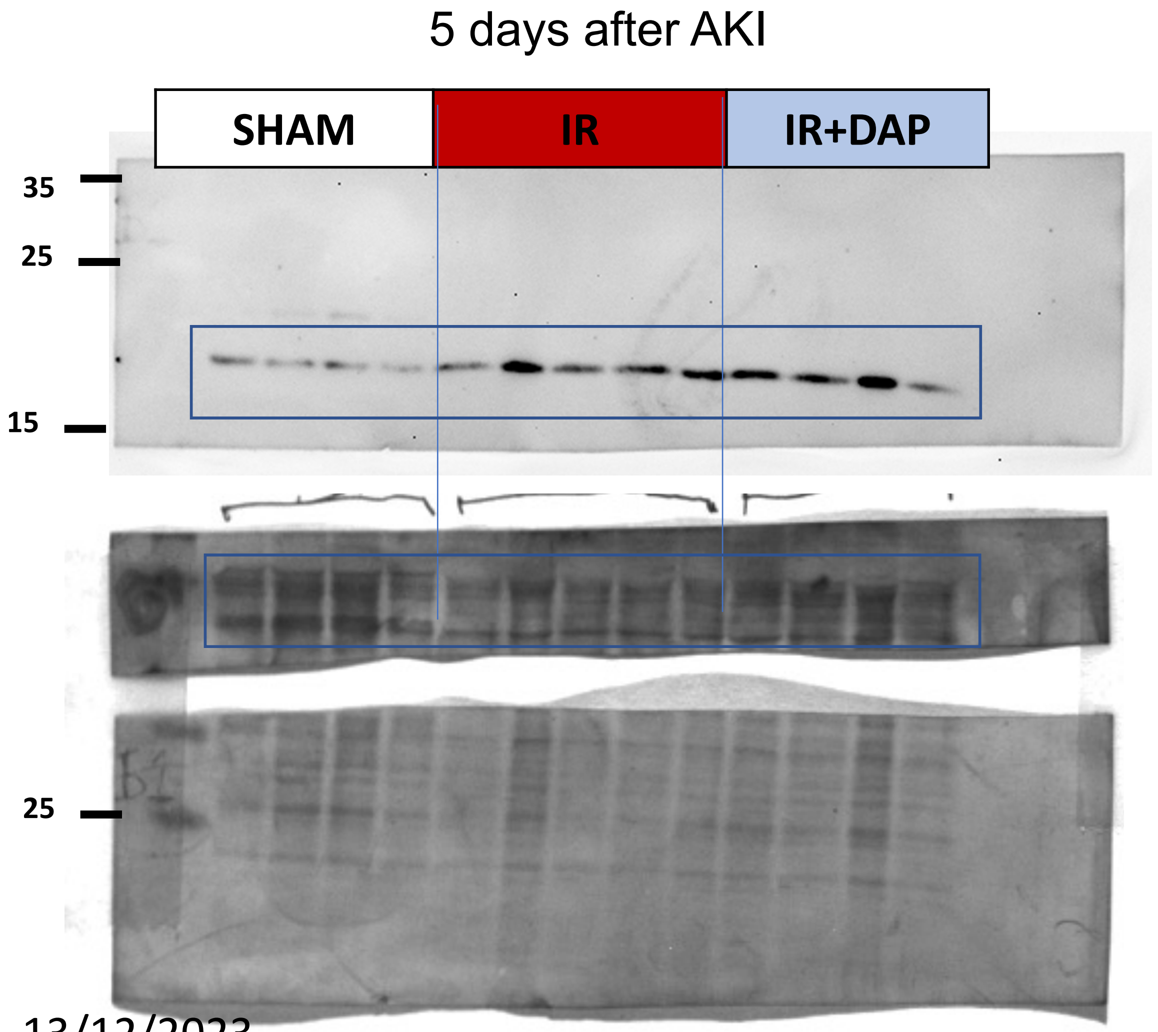

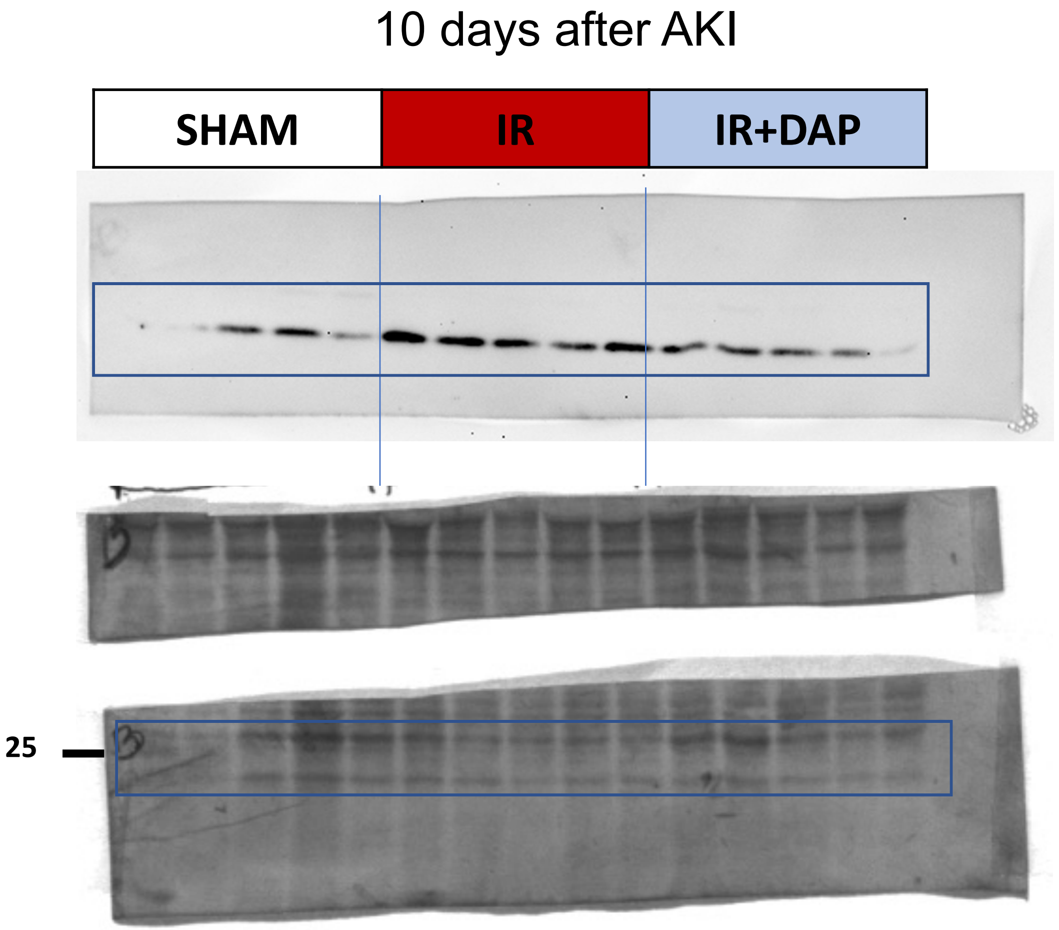

13/12/2023

Full unedited gel for Figure 6 and 8: Bnip3

60 ug of kidney cortex isolated mitochondria

Primary Mouse Anti-Bnip3 sc-56167 1:500

Anti-Mouse ab6789 1:40,000 Exp 1 min

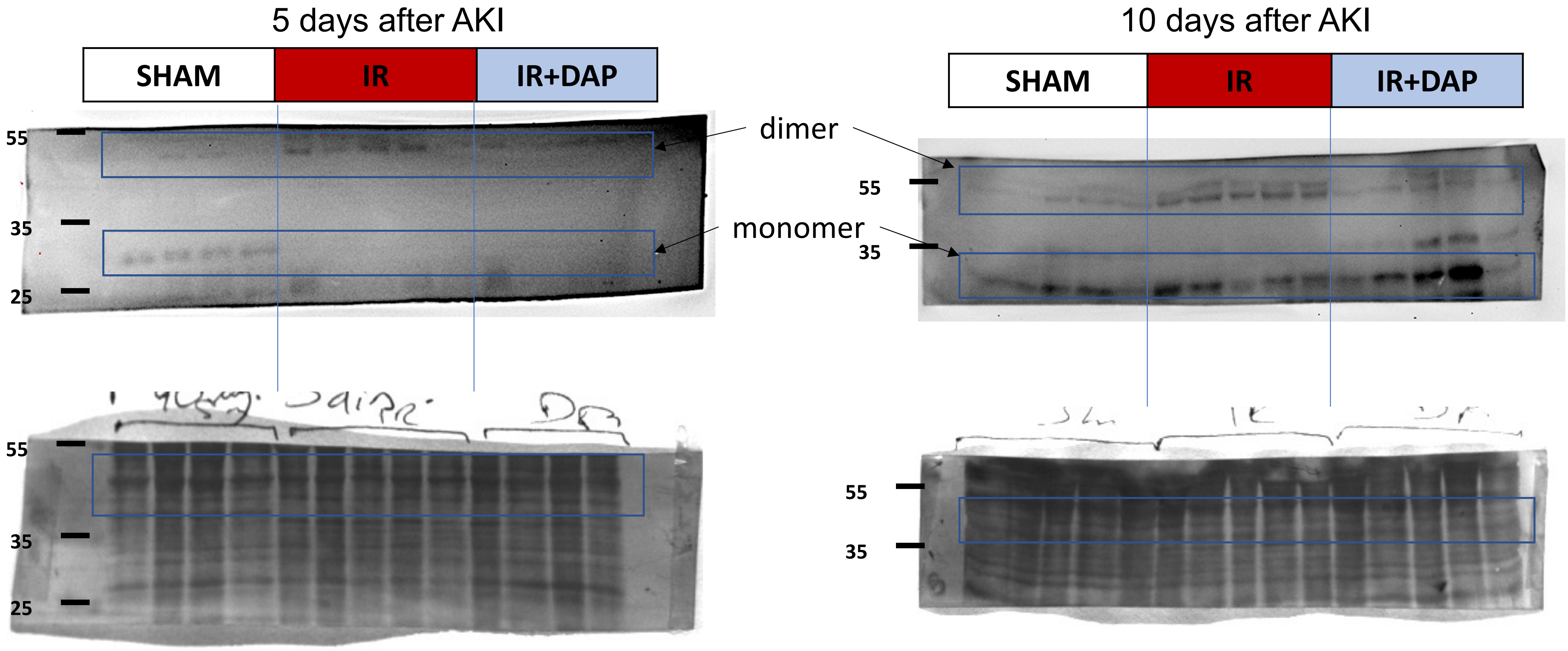

20/12/2023

Full unedited gel for Figure 6 and 8: NLRP3
20 ug of kidney cortex mitochondria

Primary Rabbit Anti-NLRP3 15101S 1:2000
Anti-Rabbit 211-032-171 1:15,000 Exp 1 min

5 days after AKI

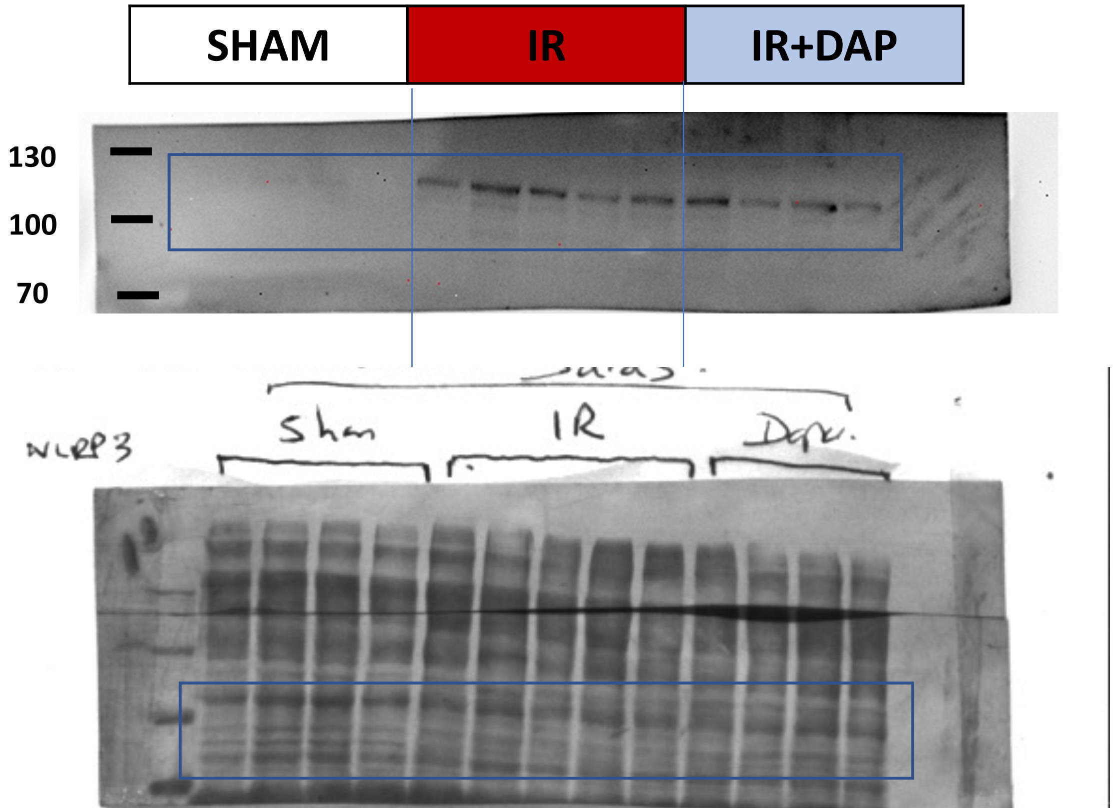

29/11/2023

10 days after AKI

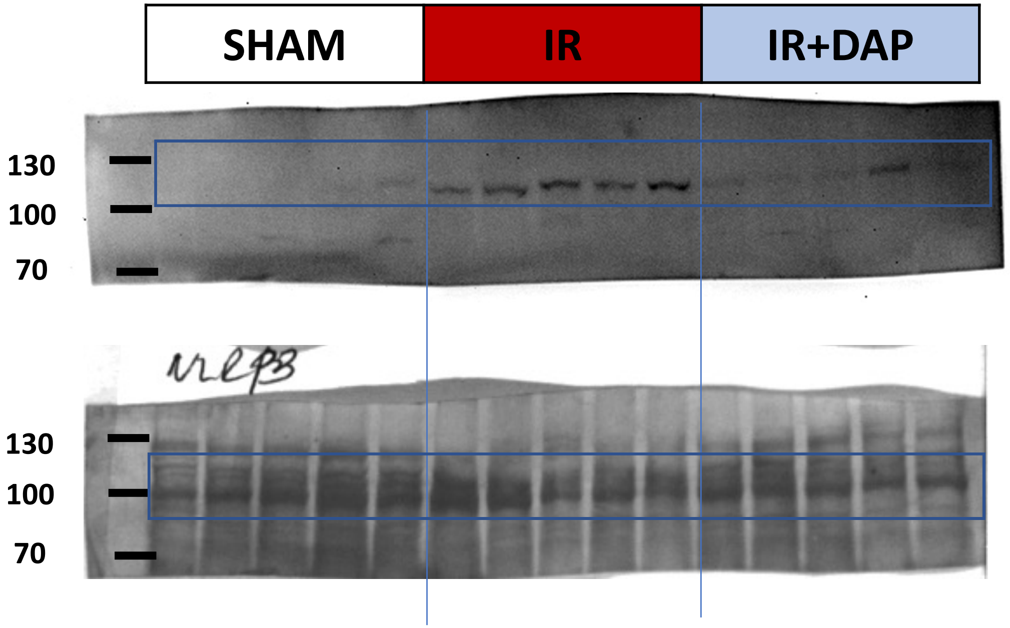

15/12/2023
